# Controlled Reduction of Genomic Heterozygosity in an Industrial Yeast Strain Reveals Wide Cryptic Phenotypic Variation

**DOI:** 10.3389/fgene.2019.00782

**Published:** 2019-09-11

**Authors:** Nadia M. V. Sampaio, Ruth A. Watson, Juan Lucas Argueso

**Affiliations:** ^1^Department of Environmental and Radiological Health Sciences, Colorado State University, Fort Collins, CO, United States; ^2^Cell and Molecular Biology Graduate Program, Colorado State University, Fort Collins, CO, United States

**Keywords:** Loss-of-Heterozygosity (LOH), *Saccharomyces cerevisiae*, industrial yeast, bioethanol, uniparental disomy

## Abstract

Abundant genomic heterozygosity can be found in wild strains of the budding yeast *Saccharomyces cerevisiae* isolated from industrial and clinical environments. The extent to which heterozygosity influences the phenotypes of these isolates is not fully understood. One such case is the PE-2/JAY270 strain, a natural hybrid widely adopted by sugarcane bioethanol distilleries for its ability to thrive under harsh biotic and abiotic stresses during industrial scale fermentation, however, it is not known whether or how the heterozygous configuration of the JAY270 genome contributes to its many desirable traits. In this study, we took a step toward exploring this question by conducting an initial functional characterization of JAY270’s heteroalleles. We manipulated the abundance and distribution of heterozygous alleles through inbreeding and targeted uniparental disomy (UPD). Unique combinations of homozygous alleles in each inbred strain revealed wide phenotypic variation for at least two important industrial traits: Heat stress tolerance and competitive growth. Quantitative trait loci analyses allowed the identification of broad genomic regions where genetic polymorphisms potentially impacted these traits, and there was no overlap between the loci associated with each. In addition, we adapted an approach to induce bidirectional UPD of three targeted pairs of chromosomes (IV, XIV, and XV), while heterozygosity was maintained elsewhere in the genome. In most cases UPD led to detectable phenotypic alterations, often in opposite directions between the two homozygous haplotypes in each UPD pair. Our results showed that both widespread and regional homozygosity could uncover cryptic phenotypic variation supported by the heteroalleles residing in the JAY270 genome. Interestingly, we characterized multiple examples of inbred and UPD strains that displayed heat tolerance or competitive growth phenotypes that were superior to their heterozygous parent. However, we propose that homozygosity for those regions may be associated with a decrease in overall fitness in the complex and dynamic distillery environment, and that may have contributed to slowing down the erosion of heterozygosity from the JAY270 genome. This study also laid a foundation for approaches that can be expanded to the identification of specific alleles of interest for industrial applications in this and other hybrid yeast strains.

## Introduction

In the budding yeast *Saccharomyces cerevisiae*, abundant heterozygosity appears to be prevalent in strains isolated from clinical and industrial settings (Borneman et al., 2011; Magwene et al., 2011; Cromie et al., 2013; Borneman et al., 2016; Peter et al., 2018). One of the first heterozygous wild strains to have its genome characterized was PE-2/JAY270 (referred to here simply as JAY270) (Argueso et al., 2009). This strain was originally isolated as an aggressive wild contaminant of sugarcane-based batch-fed fermentations (Basso et al., 2008). In addition to robust competitive growth, this strain also displays excellent fermentation yield and stress tolerance traits, thus it was selected for commercial propagation, and has since been widely adopted by bioethanol distilleries as a primary inoculum (Basso et al., 2008; Della-Bianca et al., 2013).

The industrial environment where JAY270 thrives represents an interesting model for studying the dynamics of microbial populations. During each batch of fermentation, cells are exposed to significant and variable biotic and abiotic stresses, including high osmotic pressure that transitions to ethanol toxicity, oxidative and heat stresses, and steady introduction of wild bacterial and fungal contaminants (Amorim et al., 2011). In addition, a peculiar feature of this system is that the microbial population is recycled twice daily from one batch to the next for up to eight consecutive months during the sugarcane harvest season. The combination of these factors creates a highly competitive environment, in which the most adapted yeast strains persist and may evolve over time. JAY270’s defining characteristic is its extraordinary ability to out-compete external contaminants in this environment, dominating the microbial population in the distillery and thus ensuring stable and predictable operational conditions (Basso et al., 2008).

The genetic characterization of JAY270 suggests this strain was formed as a natural hybrid that resulted from the mating of two diverged parent haploid strains (Argueso et al., 2009; Rodrigues-Prause et al., 2018). Analogous examples of such mosaic strains have been described recently, including yeasts used in the production of distilled alcoholic beverages from sugarcane juice (Barbosa et al., 2018; Legras et al., 2018; Peter et al., 2018). JAY270 is heterothallic (i.e., its meiotic spores are unable to switch mating type to self-mate and generate fully homozygous diploids), and it has a complex diploid genomic architecture, marked by abundant structural and single nucleotide polymorphisms between most pairs of homologous chromosomes (Argueso et al., 2009). This heterozygous genomic architecture is also a feature of other bioethanol strains (e.g., CAT-1, BG-1) that, like JAY270, were isolated as robust contaminants at sugarcane distilleries (Babrzadeh et al., 2012; Carvalho-Netto et al., 2013; Della-Bianca et al., 2013; Coutoune et al., 2017).

We recently mapped the distribution of heterozygous loci in JAY270 (**Figure S1**; Rodrigues-Prause et al., 2018) and found that heterozygosis is not uniformly distributed across its genome. Instead, only ∼60% of the genome corresponds to regions with a high density of heterozygous loci, interspersed by long homozygous regions. Thus, by the time this strain was isolated, ∼40% of the heterozygosis originally present in the ancestral hybrid diploid had already eroded away through cycles of mitotic and/or meiotic recombination. Presumably, the heteroalleles formerly present at those regions were likely dispensable for JAY270’s distinctive performance in the sugarcane fermentation environment. An intriguing question that follows is whether some of the heteroalleles that remain in the genome contribute to the desirable industrial traits that JAY270 displays today.

In this study, we took a step toward exploring this question by conducting an initial functional characterization of the heteroalleles present in the JAY270 genome. We employed two different approaches to reduce genomic heterozygosity, and then systematically assessed the phenotypic consequences of loss of heterozygosity (LOH). In the primary approach, we used controlled inbreeding to generate a collection of experimental strains, each harboring a unique combination of homozygous alleles distributed genome-wide. We compared the phenotypes of those inbred strains to their fully heterozygous parent (JAY270) under various culture conditions and identified candidate genomic regions where genetic polymorphisms impacted two important industrial traits: Heat stress tolerance and competitive co-culture growth kinetics. In a second, more conservative approach, we constructed strain sets in which bidirectional LOH was restricted to one chromosome pair at a time (uniparental disomy; UPD), while preserving heterozygosity elsewhere. We found that UPD also affected the two traits above, and did so in a way that was specific to the chromosomes and haplotypes that were made homozygous in each strain. Taken together, our results showed that a wide phenotypic variation can be uncovered by shuffling the combinations of heteroalleles present in JAY270. We interpreted these results in light of a model in which the current heterozygous genomic configuration of this strain may correspond to an optimal set of alleles which collectively allow it to be highly versatile, and thus well adapted to long term propagation in the industrial sugarcane fermentation environment.

## Results

### Controlled Reduction of Heterozygosity in the JAY270 Genome Through Inbreeding

In order to characterize the phenotypic contributions of the heteroalleles present in the JAY270 genome, we explored how changes in the abundance and distribution of heterozygous sites would affect the traits of the strain. Recently, we reported a draft phased map of ∼12,000 heterozygous single nucleotide polymorphisms (HetSNPs) unevenly distributed across JAY270’s genome (**Figure S1** and Rodrigues-Prause et al., 2018). In order to keep track of the two specific allele variants present at each HetSNP, we arbitrarily named the two phased haplotypes for each chromosome pair as M or P, making an analogy to haplotypes of maternal or paternal origin in a classic F1 cross (M and P alleles are represented in red and blue in all figures, respectively).

**Figure 1:**
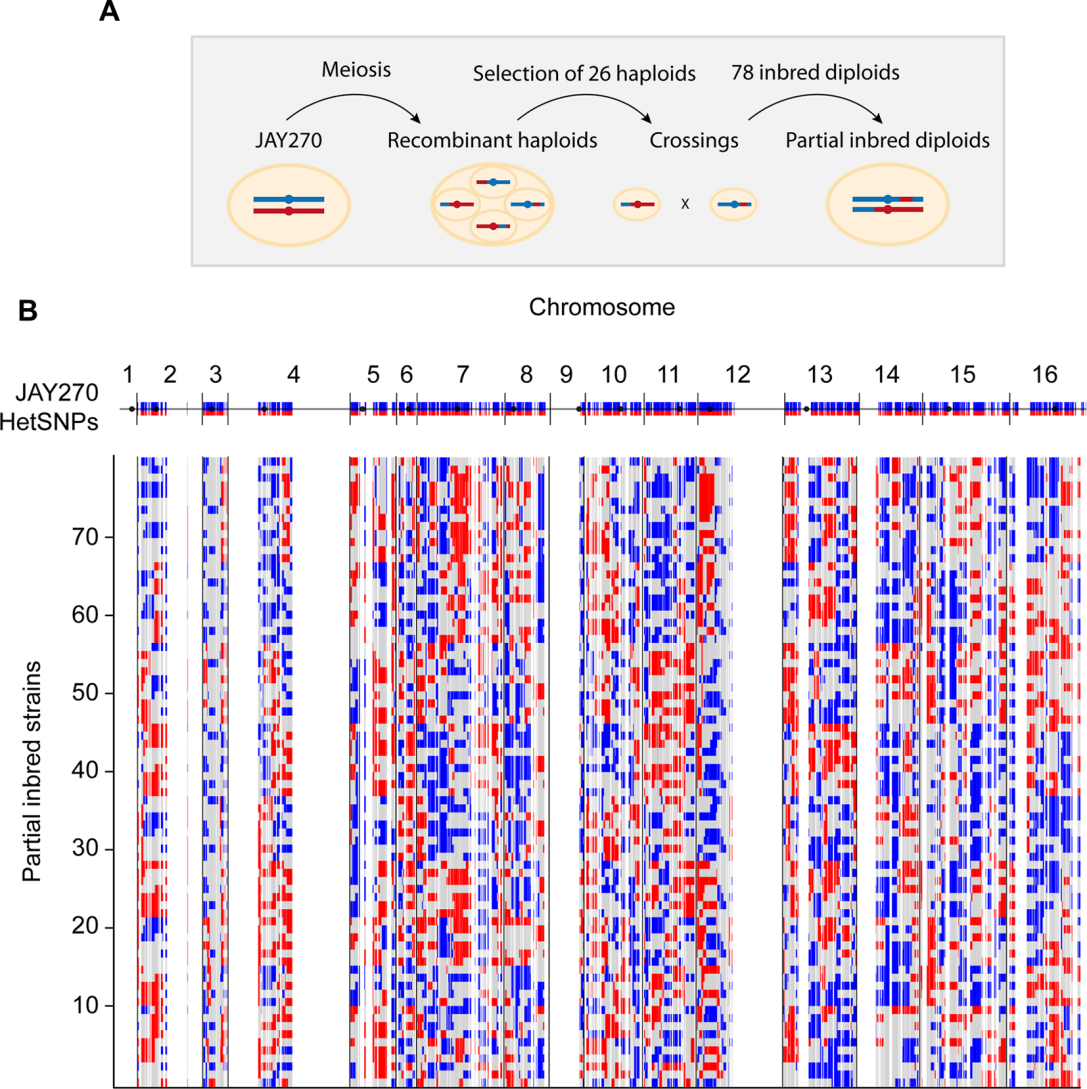
Schematic representation of the construction of inbred diploids. **(A)** A parental diploid JAY270 cell is shown with a pair of homologous chromosomes colored in blue (paternal haplotype or P) or red (maternal haplotype or M). Cells were induced to undergo meiosis, resulting in the formation of tetrads containing four recombinant haploid spores which had their whole genomes sequenced. One *MAT*α *ACE2* spore and one *MAT*a *ace2-A7* spore was selected from each of 13 tetrads and used for pairwise crossings (**Figure S2A**). This setup resulted in a collection of 78 inbred diploid strains derived from inter-tetrad matings. Each resulting inbred diploid was expected to remain heterozygous M/P at ∼50% of the genome, and to become homozygous M/M (∼25%) or P/P (∼25%) for the other half. **(B)** Genome-wide maps of the genotypes of the 78 inbred diploids. The top blue and red line corresponds to the linear representation of the distribution of 12,023 HetSNPs in the JAY270 parent genome (Rodrigues-Prause et al., 2018; **Figure S1**). Regions without blue/red lines represent genomic regions of homozygosity in JAY270. Each line below shows the maps and the distributions of genotypes in the inbred strains: Heterozygous M/P (gray); homozygous P/P (blue); homozygous M/M (red). The cumulative distribution and the overall quantification of each genotype class are shown in **Figure S2B–C**.

Our primary strategy to create JAY270 derivatives containing reduced heterozygosity was based on inbreeding. Our group had previously isolated and whole-genome sequenced 52 haploid spore clones originated from thirteen sets of JAY270 four-spored tetrads (Rodrigues-Prause et al., 2018). It has been estimated that each meiotic cell division in *S. cerevisiae* produces about 90 crossovers distributed across the genome (Mancera et al., 2008; Chakraborty et al., 2018). These events result in the formation of recombinant chromatids that are sorted into haploid spores, each containing approximately half maternal and half paternal alleles (**Figure 1A**). In order to maximize the genotypic variation of the haploids used in our crossings, we selected for mating only one *MATa* and one *MAT*α spore from each of the thirteen sequenced tetrad sets. This ensured that all inbred diploids were formed by joining recombinant haplotypes generated from independent meiotic crossover events. An additional criterion for selection of the parent spores was based on their genotype at the *ACE2* locus. We recently showed that JAY270 is heterozygous for a frameshift mutation at *ACE2* (*ace2-A7*) and diploid derivatives homozygous for the mutant allele display a cell-cell aggregation phenotype that could confound the phenotypic analysis of inbred diploids (Rodrigues-Prause et al., 2018). Thus, 13 *MATα ACE2* and 13 *MATaace2-A7* spores were crossed in inter-tetrad pairwise combinations (**Table S1** and **Figure S2A**), resulting in a collection of 78 inbred diploid strains directly derived from JAY270. This inbred collection enabled us to examine the effects of homozygosity at most regions of the JAY270 genome. The only exceptions were loci genetically linked to *MAT* and *ACE2*, respectively on chromosomes III and XII (Chr3 and Chr12), which tended to remain heterozygous in the inbred clones due to the parental haploid selection criteria described above.

Because only one generation of inbreeding was carried out, the genome of each inbred strain in the collection was predicted to be approximately a quarter homozygous for maternal alleles, a quarter homozygous for paternal alleles, and half heterozygous. Importantly, since each haploid parent inherited a unique combination of maternal and paternal alleles, no two inbred diploids were heterozygous for the same half HetSNPs. Based on the whole-genome sequence information of all 26 parental haploids, we derived precise genotype maps for each inbred diploid. These maps show all loci that remained heterozygous (M/P), and the loci that became homozygous for either allele (M/M or P/P), and illustrate the genetic variation present in our collection (**Figure 1B** and **File S1**). We analyzed the genotype maps to determine the overall level of hetero- and homozygosity in each of the inbred diploids and overall in the strain set (**Figure S2B** and **S2C**). The average inbred was heterozygous for 51% of the JAY270 HetSNPs, within a range of ∼40 to ∼62% for the least and most heterozygous inbreds. The average of M/M and P/P homozygosity was well balanced (∼26 and ∼23%, respectively) and consistent with the levels expected for a single generation of inbreeding.

### Characterization of Phenotypic Variation in the Inbred Diploid Collection

We next explored how homozygosity in each inbred diploid affected different traits in comparison to their fully heterozygous parent (JAY270). A Petri plate spotting assay format was used as an initial screen for growth phenotypes under a variety of individual stress conditions (detailed information in **Table S3**), some of which are known to be present in the sugarcane fermentation industrial environment (Della-Bianca et al., 2013). No significant changes in cell viability or growth characteristics were observed when cells were plated and grown in the presence of 7 or 11% *v/v* ethanol, 30 mM furfural (a byproduct of lignocellulose biomass fermentation), 0.75 mM of menadione (an inducer of oxidative stress), or 100 and 150 J/m^2^ ultraviolet light exposure and 0.01% methyl methanesulfonate (DNA damaging agents). The abilities to metabolize galactose and the non-fermentable carbon sources ethanol and glycerol were also apparently uniform across all inbred strains. Mild phenotypic variation was observed when cells were grown on raffinose as the sole carbon source, or in the presence of 100 mM of hydroxyurea, an inducer of DNA replication stress (data not shown).

Finally, a pronounced variation in tolerance to heat stress (growth at 39°C) was observed among strains in the inbred collection (**Figure S3**). The wide range in the distribution of this phenotype during the screening phase made it suitable for a subsequent detailed phenotypic characterization and quantitative trait loci (QTL) analysis. We categorized the inbred strains into six phenotypic groups using a qualitative colony-size scoring system (**Figure S4**). JAY270 displayed an intermediate heat tolerance phenotype (score 3), characterized by good viability, but substantial variation in colony diameter, ranging from small to medium sized colonies. Roughly 40% of the inbred strains (32 of 78) displayed a similar phenotype. The remaining inbred strains displayed heat tolerance patterns that were either lower or higher than JAY270. At the extremes were strains that either showed no growth at all or formed very small colonies when incubated at 39°C (scores 0 and 1, respectively), while others that formed uniformly large colonies and were classified as the most heat tolerant strains (score 5). The median heat stress tolerance scores for each inbred and the phenotypic distribution in the full strain set are shown in **Figure 3A**.

**Figure 2:**
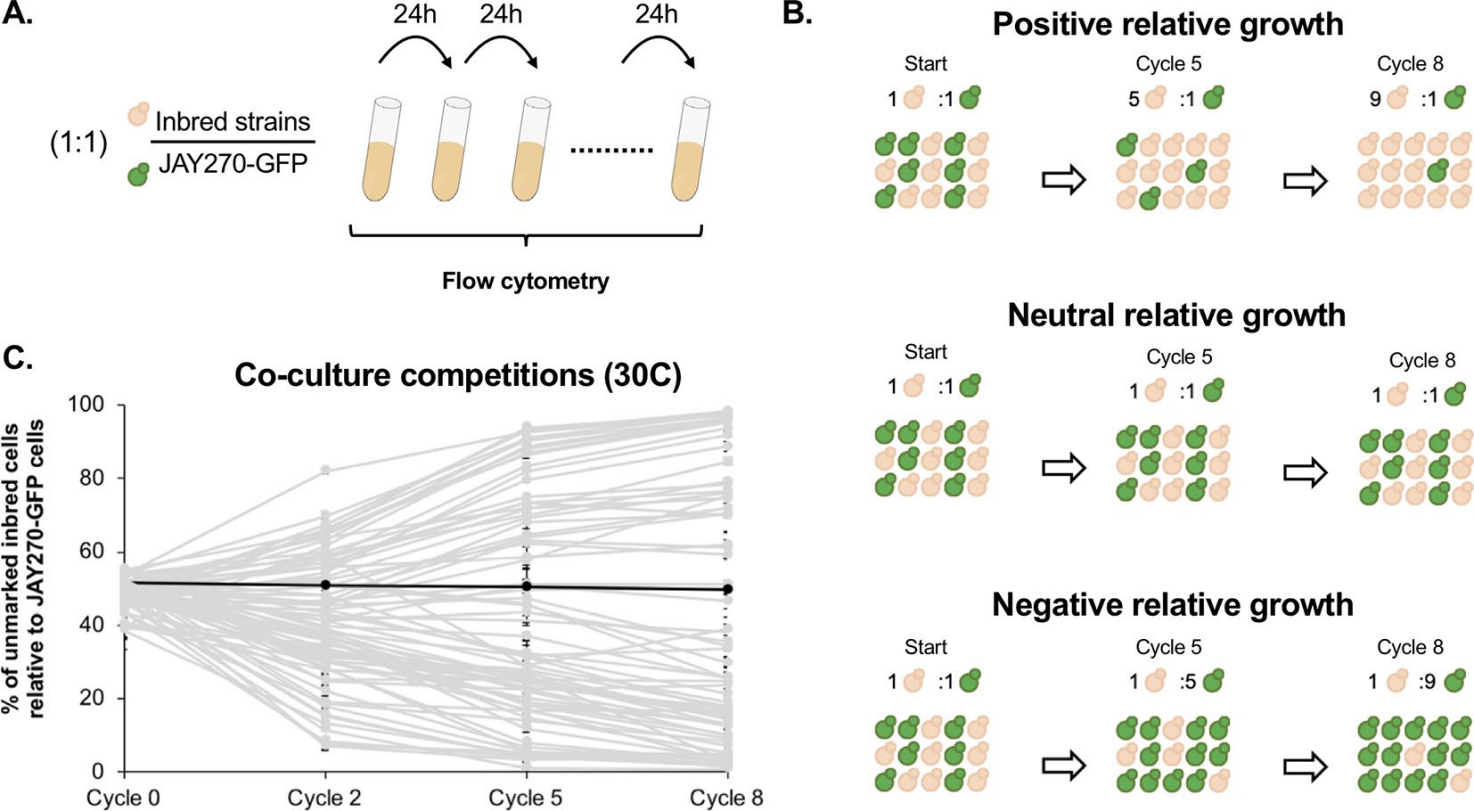
Assessment of mitotic growth kinetics through co-culture competition assays. **(A)** Co-cultures were started with an approximate 1:1 cell ratio between each unmarked inbred strain competitor (beige) and a GFP- marked JAY270 derivative (green). Every 24 h (one cycle of competition), 1% volume of each co-culture was transferred to fresh media through 8 consecutive days. The percentage of GFP- inbred and JAY270–GFP cells was monitored periodically using a flow cytometer. **(B)** Schematic representation of hypothetical inbred strains displaying positive, neutral or negative competitive growth relative to the parent JAY270–GFP. **(C)** Competitive growth profiles of the 78 inbred diploids. Each line (light gray) shows the progression of the percentage of inbred cells in the co-culture over time (average of three replicates; error bars in SD). The black line represents a control competition between the parental unmarked JAY270 and JAY270–GFP. The specific numbers used to generate the plot are detailed in **Table S4**.

In addition to the phenotypes examined through the plating assays above, we also investigated a more subtle variation in mitotic growth kinetics. JAY270 is known to grow very robustly, and this trait is likely a key factor contributing to its ability to outcompete wild yeast contaminants in the sugarcane fermentation process. Thus, we sought to explore the variation in the growth kinetics phenotype among the inbred strains through a cumulative co-culture competition assay (**Figure 2A**). Each inbred strain was co-cultured with a GFP-marked JAY270 derivative (JAY270-GFP) under optimal *S. cerevisiae* growth conditions (YPD liquid rich medium at 30°C under rotation). The co-cultures were started with an approximately equal inoculum of the two competitors (∼2.5 x 10^6^ cells each) and were incubated for 24 h, past nutrient depletion and population saturation (∼15 h). At the end of each daily growth cycle, 1% volume of each co-culture was transferred to fresh liquid medium to allow continued growth. The percentage of GFP-negative (inbreds) and GFP-positive (fully heterozygous JAY270) present in the co-cultures was measured periodically with a high-throughput flow cytometer (**Figure S5A–C**), and used as a parameter to quantify the growth kinetics of each of the inbred diploids relative to JAY270. Inbred strains with intact growth kinetics should maintain steady ∼50% over time, whereas deviations up or down would indicate a phenotypic change (**Figure 2B**).

Besides the genotype of the inbreds, another factor that may cause the relative abundance of the GFP− and GFP+ competitors to deviate is the emergence of beneficial *de novo* mutations within the co-cultures. However, this effect should be delayed until the newly formed mutants become numerous enough to be detected. In order to determine the period of time during which the GFP− to GFP+ ratio can be confidently attributed solely to the initial genotype of the inbreds, we performed control co-culture competitions of each of four independently generated GFP− marked JAY270 clones versus the unmarked JAY270 parent strain. We carried out a total of twelve co-cultures (four GFP− marked clones, three replicates each) with daily 1% volume transfer cycles to fresh media for 22 consecutive days, and the percentage of GFP− was measured at 7-day intervals (**Figure S5D**). The GFP ratios in all 12 independent co-cultures remained steady at ∼50%:∼50% by the end of the first week (cycle 8). By the end of the second and third weeks, some of the ratios had diverged up or down, presumably through emergence of beneficial mutations in the GFP− or GFP+ strains. Therefore, we limited our experimental competitions of the inbred diploids versus JAY270–GFP to a maximum of 8 daily transfer cycles, in order to insulate the measured GFP ratios from the effect of *de novo* mutations. These control experiments also showed that integration of the GFP cassette into the JAY270 genome did not by itself have an effect on growth kinetics. Additional JAY270–GFP versus JAY270 control co-culture competitions were included every time a new experimental evaluation of the inbred collection was performed (39 replicates), and in no cases a significant deviation in the GFP ratio was observed before or at transfer cycle 8.

The competitive growth profiles of the inbreds were characterized collectively by a “fan out” shape, showing that a wide range of phenotypic variation existed in the strain set (**Figure 2C**). Many of the inbred diploids displayed growth kinetics that were substantially different from JAY270, not only slower but also faster. Of this group at the extremes of the competitiveness range, 18 displayed a strong reduction in growth kinetics and comprised less than 10% of the total cell population by the last cycle of co-culture; while 13 inbreds showed a substantial improvement in growth, outcompeting JAY270 to reach more than 90% of the cells in the co-cultures. Importantly, all but one of the inbreds, regardless of the neutral, positive or negative relative growth kinetics profiles, followed a steady unidirectional trajectory from the early cycles until the end of co-culture. This result is consistent with their phenotype being a function of their initial genotype, and not due to the random appearance of *de novo* mutations during the experiments. In addition, there was very little variation between the independent replicates of each inbred co-culture, further disfavoring a potential influence of *de novo* mutations over the observed phenotypes.

**Figure 3:**
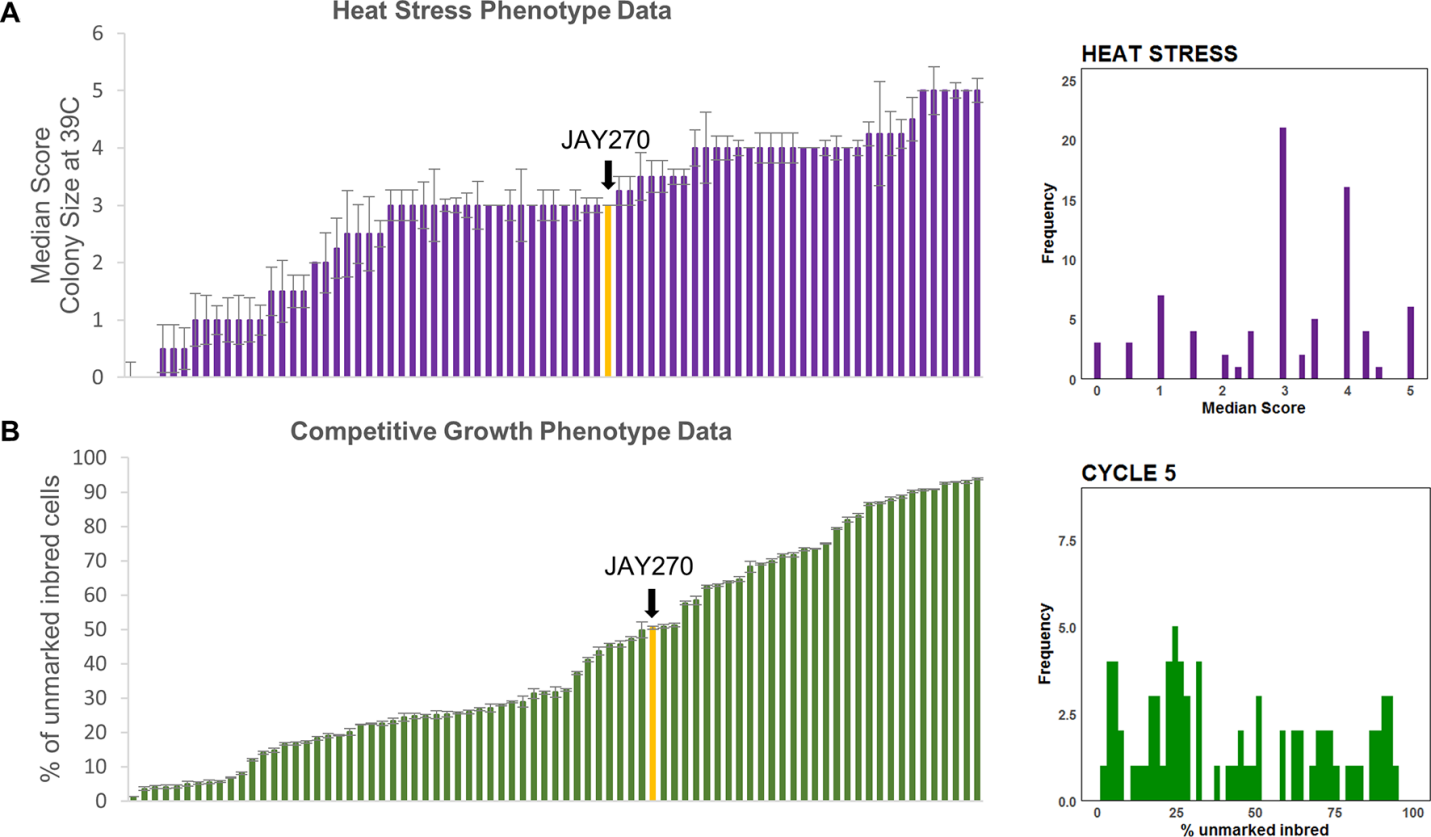
Phenotypic distributions in the inbred strain collection. **(A)** Left side: Median (error bars in standard error of the median) heat tolerance scores for inbred strains, ordered from lowest to highest; Right side: Frequency distribution of the phenotypic values shown to the left. **(B)** Left side: Mean (error bars in standard error) of competitive growth for inbred strains (% unmarked cells at cycle 5), ordered from lowest to highest; Right side: Frequency distribution of the phenotypic values shown to the left. In both plots the bars corresponding to the JAY270 phenotype are highlighted in yellow and indicated by a black arrow.

It is important to note that all 78 inbreds, even those with the poorest performance in the co-culture competition, grew apparently normally and indistinguishably from JAY270 on solid/agar rich medium at 30°C. This shows that the cumulative liquid co-culture competition assay was able to reliably and consistently uncover extremely subtle relative differences in growth kinetics. We estimate that the most extreme competition phenotypes among the inbreds, reaching <10% or >90% of the total co-culture cell population by transfer cycle 8, should have a rate of cell division only ∼3% longer or shorter than JAY270, respectively. Thus, the co-culture competition assay offered an opportunity to reliably measure minor phenotypic changes that resulted from the different genotype combinations represented in the inbred collection. Even though we collected data for cycles 0, 2, 5, and 8, we used data only from cycle 5 for the downstream QTL analysis as it offered an optimal quantification of relative growth kinetics. The cumulative nature of this assay meant that by cycle 8 some of the extreme GFP ratios had already started to reach a plateau, which could lead to an underestimation of their full phenotypic differential. This cumulative trend can be visualized in **Figure S6** as the progressive shift in the phenotypic distribution away from center (cycles 0 and 2) and toward the low and high extremes over time (cycle 8). The specific mean percentage of each inbred in the co-culture at cycle 5 and the phenotypic distribution in the full strain set are shown in **Figure 3B**.

### Identification of Genomic Regions Associated With Phenotypic Variation

**Figure 4:**
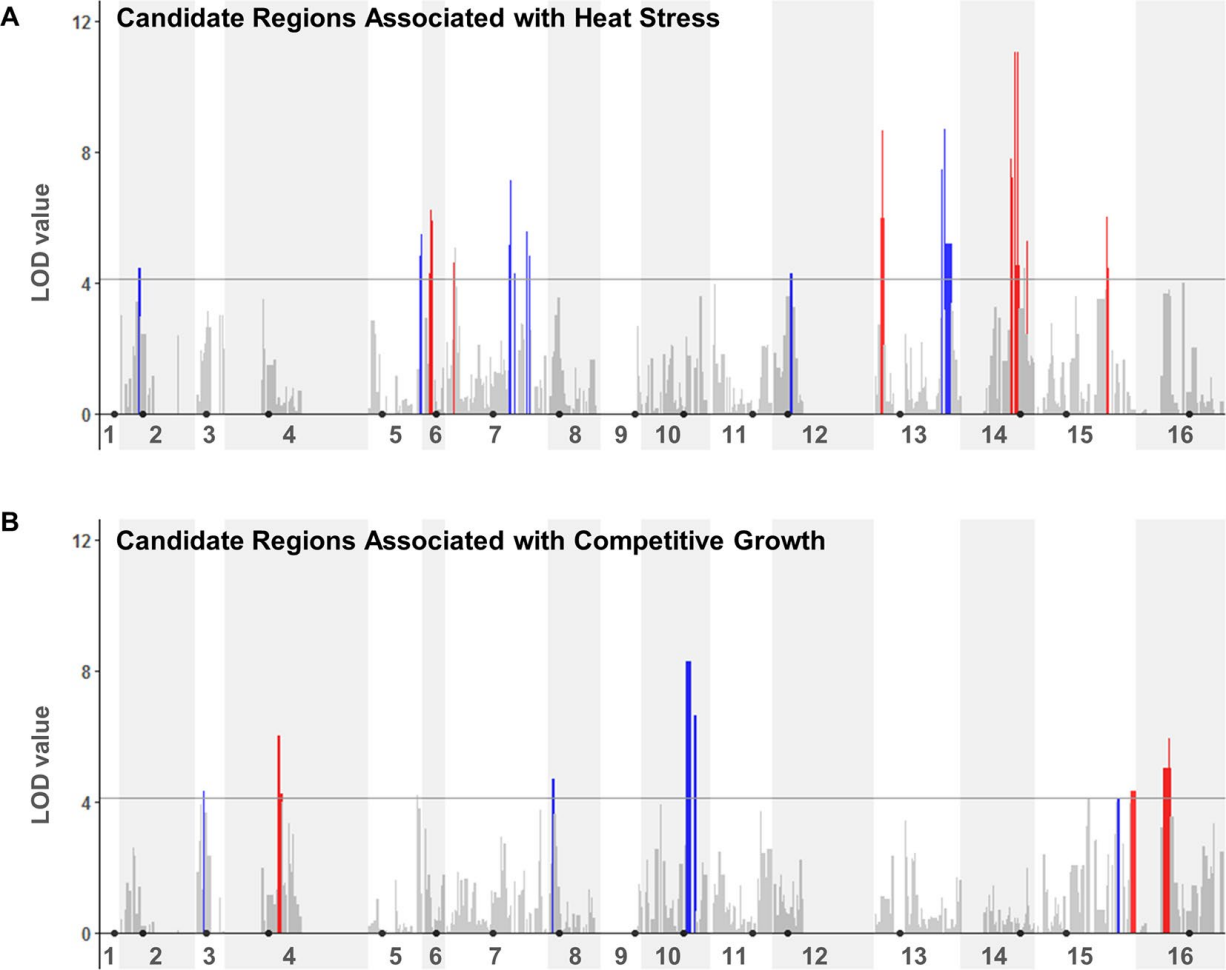
QTL analyses to identify loci potentially underlying heat stress tolerance **(A)** and relative growth kinetics **(B)**. LOD scores for each HetSNP (y-axis) were plotted over linear genomic coordinates of the HetSNPs for all 16 chromosomes (x-axis). The horizontal gray line corresponds to the LOD = 4.11 threshold of significant association. The color code in the plots indicates the allele contributing the higher phenotype value (red: maternal; blue: paternal). Black circles indicate the positions of centromeres. Plots were generated in R (version 3.4.0) using the ggplot2 package (version 1.42-8) and a custom script.

We next performed a QTL analysis to identify possible relationships between the specific genotypes at JAY270 HetSNPs and the phenotypic variation in heat stress tolerance and competitive growth among inbred strains. Because all the inbred diploids in the collection were necessarily heterozygous at Chr3 *MAT* (*MATa*/*MAT*α) and at Chr12 *ACE2* (*ACE2*/*ace2-A7*), we excluded markers genetically linked to those loci from the analyses (within ∼50 and ∼75 Kb up and downstream of each, respectively). This resulted in a final list of 11,742 HetSNPs that were included in the QTL analyses. We used the genotype maps of all inbreds (**File S1**) to determine the frequencies of homozygous M/M and P/P, and heterozygous M/P genotypes at each HetSNP marker. Then, for each marker we calculated the mean phenotype value measured among strains with M/M, P/P and M/P genotypes. Using a one-dimensional scan of the genome, log10 likelihood ratio (LOD) scores were determined for each marker for each trait. The statistical significance thresholds for the identification of candidate loci associated to each trait were established by randomized phenotype by genotype permutation tests (five independent runs of 10,000 iterations for each trait) at the *p* < 0.05 significance level. The significance threshold values determined independently from the heat tolerance and competitive growth permutation tests were the same: LOD > 4.11. The genome-wide LOD scores for each trait are plotted in **Figure 4**, and regions that rose above the 4.11 thresholds were considered to be statistically significant. A two-dimensional scan of the genome was also performed, but no significant pairwise epistatic interactions were detected (data not shown).

Although our inbred population size was relatively small, this analysis was sufficient to reveal multiple genomic segments that may make important contributions to the traits of interest. In total, thirteen regions from eight chromosomes showed association to heat tolerance, and seven regions from six chromosomes to competitive growth (**Table S6**). For each trait, these regions corresponded to combined total sizes of ∼332 Kb with ∼189 annotated genes in them, ∼25% of which were heterozygous for non-synonymous substitutions. This narrower list included 47 and 48 candidate genes within the genomic regions that were significantly associated to heat tolerance and competitive growth, respectively. The genes in both lists belonged to diverse functional annotation groups (i.e., no specific Gene Ontology terms were significantly enriched at *p* < 0.01).

We evaluated which quantitative inheritance model better fit the observations from each region (**Table S7**). Most regions (16 of 20) were consistent with an additive variance model in which the heterozygote has an intermediate phenotype. We also found four regions with likely dominance, but no cases of overdominance. Finally, we estimated the percent variance explained (PVE; **Table S7**) for the HetSNP with the highest LOD value within each region using a single-QTL model analysis. In order to facilitate a comparison of the relative contributions between regions to each trait, we also calculated relative PVE values normalized to the locus with the highest PVE. As an additional approach, we fit a multi-QTL model (**Table S8**) and determined that three of the regions identified for heat tolerance synergistically explained 76% of the variance and three of the regions for competitive growth worked together to explain 57% of the variance.

The identification and characterization of specific major genes and alleles that contribute to these traits in JAY270 was beyond the scope of this particular study. However, we noted that none of the significant association regions overlapped between the two traits. In addition, there was no overlap between the inbred strains ranked in the upper or lower tiers of heat tolerance and competitive growth (**Figure S7**). This suggested that the two traits are controlled independently of each other, so different combinations of alleles present at different sets of JAY270 genomic regions contributed in their own way to the phenotypic variation observed for each trait.

### Controlled Reduction of Heterozygosity in the JAY270 Genome Through Targeted Uniparental Disomy

In the inbred collection approach described above, each strain had lost roughly half of the heterozygosis present in JAY270, thus a large fraction of the genome was affected. We next took an independent and more conservative approach in which fewer heterozygous loci were manipulated at a time. To do so, we adapted a procedure to induce targeted uniparental disomy (UPD) (i.e., homozygosis for an individual whole chromosome), while preserving heterozygosis in the other chromosome pairs. Our strategy took advantage of previous demonstrations that driving transcription through centromeric regions leads to perturbation of the function of centromeres, and can be used to induce targeted chromosome loss, resulting in 2*n* − 1 monosomic diploid cells (Hill and Bloom, 1987). This strategy was successfully applied to map mutations to individual chromosomes in a *ura3*/*ura3* auxotrophic diploid laboratory strain background (W303; (Reid et al., 2008)), by inducing transcription of a p*GAL1-URA3* cassette integrated at centromeric regions, and then applying counter selection for 5-FOA resistance to recover clones that had lost the targeted chromosomes.

**Figure 5:**
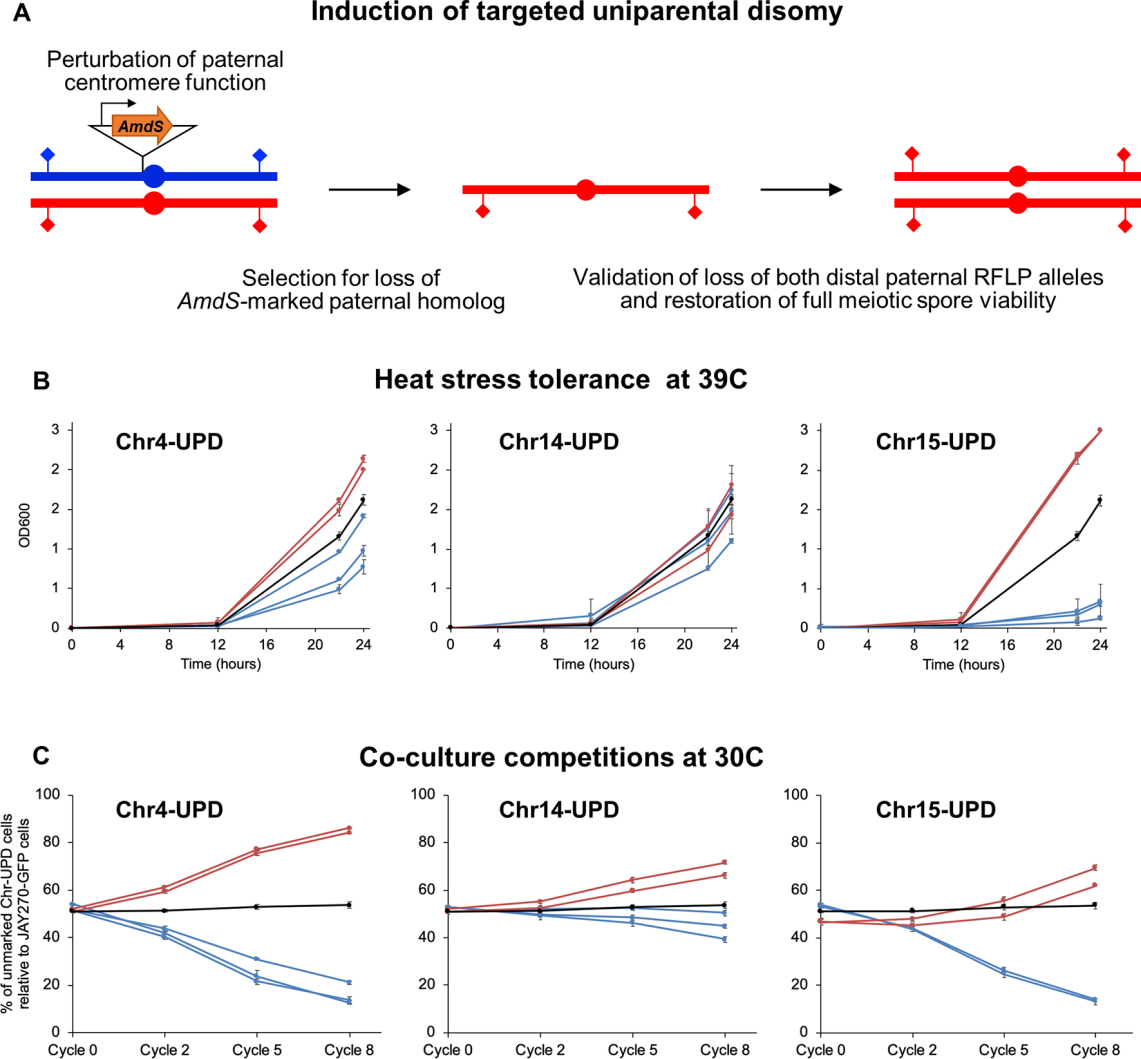
Construction and phenotypes of UPD strain pairs. **(A)** A cassette containing the counter-selectable marker *AmdS* under the transcriptional control of the *TEF1* constitutive promoter and lacking a terminator sequence was integrated immediately upstream of the centromeric regions of each M or P homolog of Chr4, Chr14, and Chr15 (insertion of *AmdS* at a P homolog is shown in this case). Transcription of *AmdS* perturbs centromere function and induces targeted chromosome mis-segregation during mitosis. Cells that lost the *AmdS* marker were selected for in media containing fluoroacetamide. Spontaneous endoduplication of the remaining homolog results in strains containing UPD, in this case represented as the maternal (red) homolog. Loss of each homolog was validated by RFLP-PCR genotyping analysis at distal markers on both chromosome arms (diamonds), and confirmation of endoduplication was obtained by tetrad dissection and spore viability analysis (4 viable spores per tetrad indicate disomy). Multiple independently-generated UPD strains were isolated and used in phenotypic tests. **(B)** Growth profiles under heat stress of UPD strain pairs. Each line shows the growth curves of an individual UPD strain under high temperature conditions (39°C) in liquid culture; error bars in SD. The OD600 at 0, 12, 22, and 24 h are shown on the y-axis. Red and blue lines represent strains containing two copies of the maternal (M/M) or paternal (P/P) homologs, respectively, of each chromosome analyzed (Chr4, Chr14, and Chr15). The black line represents the JAY270 control. **(C)** Competitive growth profiles of UPD strain pairs. Each line shows the relative growth profile of an individual UPD strain in co-culture with JAY270–GFP, grown at 30°C; error bars in SD. The percentage of UPD cells relative to JAY270–GFP cells at the 0, 2, 5 and 8^th^ cycle of co-culture are shown in the y-axis. Color scheme is the same as in B. The black line corresponds to a control competition between the parental unmarked JAY270 and JAY270–GFP.

Here, we adapted this approach for use in prototrophic diploid strains by integrating a hemizygous copy of the heterologous forward and counter selectable marker *AmdS* (Solis-Escalante et al., 2013) immediately upstream of specific JAY270 centromeric regions. We modified the *AmdS* cassette by removing the transcriptional terminator sequence, thus enabling constitutive transcription to continue past the ORF and extend through the centromeric sequence. Insertions of *AmdS* cassettes adjacent to centromeres of each the M and P homologs of targeted chromosomes were obtained and stably maintained through forward selection for growth in media containing acetamide as the sole nitrogen source (**Figure 5A**). Then, counter selection for loss of the cassette (fluoroacetamide resistance) was used to isolate candidate clones carrying chromosome loss. The final phase, and a key part of the strategy, relied on the observation that monosomic diploid *S. cerevisiae* cells tend to rapidly and spontaneously endoduplicate the remaining homolog, which results in reestablishment of the normal chromosomal complement through UPD (Reid et al., 2008). Another possible mechanism is that UPD may be formed in a single step through meiosis I-like co-segregation of sister chromatids in mitotic cells (Andersen and Petes, 2012).

We conducted a proof-of-concept experiment focused on the generation of strain pairs carrying bidirectional UPD for three chromosomes (Chr4, Chr14, and Chr15), chosen on the basis of their overall chromosome size, and number and distribution of HetSNPs (**Figure S1**). It has been proposed that loss of long *S. cerevisiae* chromosomes may impose a heavier phenotypic burden than loss of a small chromosome (Reid et al., 2008), thus the likelihood to recover UPD through endoduplication should be higher. Chr4 was an attractive candidate for this analysis because it is a large chromosome but has a relatively low number of HetSNPs, which are all clustered in a central region. We also chose to study Chr15, because it is also a large chromosome, but in contrast to Chr4 it has a large number of HetSNPs (> 1,400; ∼12% of the genome’s total) scattered throughout its whole length. Finally, a third interesting case study was Chr14, which is a mid-size chromosome containing a relatively large number of HetSNPs (∼700) and a long homozygous segment.

We integrated the terminator-less *AmdS* cassette immediately adjacent to the centromeres of each homolog of these three chromosomes. We then screened multiple independently-generated chromosome loss clones by PCR-RFLP genotyping followed by tetrad analysis to identify those that had undergone UPD to become homozygous for each of the three respective targeted chromosomes (**Figure S8**). We tested by tetrad analysis a subset of the fluoroacetamide resistant clones that had LOH at both the left and right centromere-distal HetSNP markers. All of the clones tested through tetrad analysis were found to be disomic (*i.e*., four viable meiotic spores per tetrad; data not shown), indicating that monosomy of Chr4, Chr14, and Chr15 was short-lived, and UPD was readily acquired. As an additional control, we determined the genotypes of all UPD strains at three heterozygous loci that were not targeted for UPD (**Figure S9**). All strains remained heterozygous at those loci, except for the specific chromosome homolog targeted for UPD.

### Phenotypic Consequences of Chromosome-Scale LOH

Our goal was to test whether localized reduction of heterozygosity in these three chromosomes would be sufficient to cause detectable variations in the heat tolerance and competitive growth phenotypes. It is important to note that, if UPD induction indeed led to phenotypic changes, we would not necessarily expect those to correlate with the presence of significant peaks identified through the QTL analyses derived from phenotypes of inbred strains. The association of the HetSNPs present in those recombinant chromosomes likely reflects complex interactions with the homo- and heterozygous genotypes of loci present in other regions of the genome. In contrast, any phenotypes detectable in the UPD strains would likely be dependent on the collective and coordinated homozygosity of all alleles present in each M or P haplotypes for the respective chromosome homologs, within a background of heterozygosis everywhere else.

Earlier in the study we used a colony size qualitative scoring system to describe the variation in the heat tolerance phenotype among the strains in the inbred collection. In order to improve the characterization of more subtle phenotypic differences among the UPD strains, we monitored optical density (OD600) in pure cultures in liquid media under rotation at 39°C. We validated this approach by generating 39°C pure culture liquid growth curves for JAY270, and for two heat tolerant and two heat sensitive inbreds, and compared them to the results of parallel solid media qualitative colony scoring assays of these same control strains. The heat tolerance profiles for each of the strains were quite consistent between the two assays (**Figure S10**). The liquid growth assay is more laborious and thus less suitable for the analysis of large strain sets (i.e., the whole inbred collection). However, it provided more informative data because its broader dynamic range allowed us to better monitor refined gradations of the heat stress tolerance phenotype.

Using this enhanced method (**Figure 5B**), we found that UPD for the two Chr15 haplotypes influenced heat tolerance significantly and in opposite directions. Chr15-UPD M/M strains were more heat tolerant than JAY270, while Chr15-UPD P/P strains were quite sensitive. A similar, but less pronounced pattern was observed for Chr4-UPD M/M and Chr4-UPD P/P, which showed, respectively, slightly higher and lower tolerance to heat stress compared to JAY270. Finally, Chr14-UPD in either direction did not appear to cause substantial difference in heat tolerance.

Co-culture growth competition assays also revealed pronounced phenotypic shifts in the UPD strains (**Figure 5C**). Homozygosis for the two Chr4 haplotypes influenced the growth kinetics significantly and in opposite directions, in a symmetric fashion. Chr4-UPD M/M strains outgrew the fully heterozygous parent JAY270, whereas Chr4-UDP P/P strains displayed the opposite phenotype. Homozygosis for the two Chr15 haplotypes resulted in a less symmetrical change in growth kinetics, but still followed a similar trend in which each haplotype displayed opposite competition profiles. Chr15-UPD M/M strains displayed a subtle but steady growth advantage, while Chr15-UPD P/P strains were outcompeted by the parent strain JAY270 at a faster pace. Finally, the changes in growth competition profiles of the Chr14-UPD strains were more subtle, but also showed a divergent trend between haplotypes.

Notably, all independently-generated strains within each of the six UPD sets displayed very similar phenotypes for both heat tolerance and competitive growth. This indicated that any non-targeted alterations that may have arisen in their genomes during construction, if at all present, were not sufficient to influence these phenotypes. Taken together, our results showed that even though each of the three UPD pairs retained ∼88–96% of the overall HetSNPs of JAY270, their relatively small and localized erosions of heterozygosis were sufficient to create significant and often symmetric alterations in the two phenotypes examined.

## Discussion

The work presented above showed that the heterozygous genome of JAY270 harbors a diversity of alleles that can support a wide phenotypic variation for competitive growth and heat stress tolerance. Our QTL analyses using inbred diploids pointed to broad regions scattered throughout the genome that were associated with these two traits. The genomic regions identified in each case were distinct between them (**Figure 4** and **Table S6**), and the groups of inbred diploids ranked at the top and bottom of each phenotypic range were non-overlapping (**Figure S7**). Interestingly, we found that the inbred clones displayed superior just as often as inferior performances compared to the heterozygous JAY270 parent strain for the two narrowly defined traits analyzed.

The phenotypic analysis of strains engineered for carrying bidirectional UPD of targeted chromosomes also provided important clues about the extent to which heterozygosity influences the overall phenotypes of JAY270. The cumulative effects of homozygosis in entire chromosomes resulted in detectable changes in both heat stress tolerance and competitive growth (**Figure 5**). Homozygosis for each haplotype within a chromosome often led to opposite phenotypic outcomes, characterized mostly by a symmetric response relative to the heterozygous parent. This pattern was especially noticeable in the Chr4-UPD strains in growth kinetics and the Chr15-UPD strains in heat tolerance. In these two cases, the M/M UPD derivatives were superior to JAY270 while the P/P derivatives were inferior. However, it is entirely possible, and we believe likely, that other phenotypes not specifically tested in this study could have diverged in opposite directions such that P/P UPDs would be superior to JAY270 and M/M UPDs.

The cryptic phenotypic variation uncovered in the inbred and UPD strain sets engineered for this study may also be accessible naturally to the JAY270 lineage during industrial fermentations. This may be achieved quickly and globally through meiotic recombination followed by inbreeding, and/or gradually and locally through mitotic recombination (Magwene, 2014; Dutta et al., 2017). Assuming that the ancestor of the lineage that gave rise to JAY270 was a hybrid diploid formed by mating of two diverged haploids, it would have been heterozygous for loci distributed across 100% of its genome. One cycle of meiosis in that ancestor followed by mating between sibling spores from the same tetrad would produce a diploid bearing only ∼66% of the heterozygosity originally present in the ancestor (Johnson et al., 2004), or ∼50% heterozygosity if the mating occurred between spores from two different tetrads (**Figure S2**). A single such cycle of meiosis is sufficient to explain most or all of the distribution of heterozygosity observed in the JAY270 genome (∼60%; **Figure S1**). Two or more meiotic cycles over the life history of this lineage would result in substantially less heterozygous diploids (∼44% or less). The second and more gradual path to the erosion of the heterozygosity originally present in the hybrid ancestor would be multiple rounds of allelic inter-homolog mitotic recombination over successive generations of vegetative growth. This would lead to the progressive accumulation of many tracts of homozygosity distributed genome-wide. Either mechanism, or a combination of them, may have contributed to shaping the JAY270 genome. However, we favor a predominant role for mitotic recombination, given the long record of propagation of the PE-2 industrial strain (Basso et al., 2008), from which JAY270 was purified (Argueso et al., 2009).

In many species inbreeding is known to correlate with inferior phenotypes and decreased fitness (Charlesworth and Willis, 2009). In contrast, in this study we described multiple cases in which homozygosity resulted in superior performance. However, it is essential to note that neither of the individual and narrow phenotypic assays we used reproduced, nor approached, the complex and dynamic sugarcane fermentation environment that likely shaped the present genomic configuration of JAY270. If it had been possible for us to exactly reproduce the biotic and abiotic challenges found in sugarcane bioethanol distilleries, then we predict that most inbred and UPD clones would perform poorly relative to JAY270, and few or none would be superior. The challenges posed by such varied and simultaneous stress conditions might be better met by the heterozygous genomic configuration that enables JAY270 to be a well-rounded generalist, the feature that makes it so attractive to bioethanol producers.

We interpret our results within the context of a model in which the erosion of heterozygosity in the JAY270 genome through meiotic and/or mitotic recombination, while frequent and potentially beneficial in specific circumstances, may be generally disfavored and curtailed by natural selection. For example, homozygosis at a specific chromosomal region might lead to faster growth, but it may also decrease tolerance to elevated temperatures or other unrelated stress sensitivities. Once cells carrying such new LOH tracts arise, selective pressures to which the cells are subjected to in the distillery environment would determine their fate of expansion or disappearance from the yeast population. The adaptive potential of LOH has been nicely characterized in inter- and intra-species yeast hybrids grown in chemostats over several generations under different and specific growth conditions (Smukowski Heil et al., 2017). Mitotic recombination leading to LOH was shown to be a major driver of adaptation in those hybrids. However, when clones carrying an LOH event that conferred superior fitness in a specific growth condition were tested in an alternate condition, their fitness was often reduced. This is consistent with our findings that a genome-wide or even regional reduction in heterozygosity through inbreeding or UPD can have positive effects for some specific traits, but may not necessarily support overall fitness.

We speculate that the net result of such LOH events in JAY270 would often be disadvantageous in its natural environment where optimal performance is constantly demanded for all phenotypes. This opposing interaction between LOH steadily introduced by recombination versus selection for optimal adaptation to a complex and dynamic environment could explain the persistence of heterozygous regions in the JAY270 genome. Given that LOH events occur at a substantial rate in yeast genomes and that JAY270 had been clonally propagated at industrial scale for years prior to isolation (and perhaps longer in natural environments), we reason that most of its genomic heterozygosis could have already been eroded away. However, the fact that a substantial portion (∼60%) of the JAY270 genome still retains heterozygosity suggests that an opposing force (*i.e*. natural selection) might have acted to disfavor cells carrying LOH spanning loci and heteroalleles that contribute to the strain’s overall fitness.

Here, we leveraged inbreeding and induction of targeted UPD to characterize the phenotypic consequences of controlled reductions in the levels of genomic heterozygosity in a natural hybrid yeast strain. Our results using both approaches revealed a wide phenotypic variability provided by the HetSNPs distributed through the JAY270 genome, and allowed the initial identification of broad genomic regions associated with two important industrial traits. This study laid the foundation for future experimental work aimed at refining these maps to identify specific heteroalleles. A higher resolution of genetic mapping can likely be achieved by substantially increasing the size of the inbred collection, by generating UPD strain pairs for the remaining thirteen chromosomes, and also by targeting mitotic recombination and LOH to specific chromosomal regions of interest (Sadhu et al., 2016). Coupling these expanded strain sets with automated high throughput phenotyping for a wider variety of growth conditions might allow identification of individual heteroalleles and loci associated with traits of interest for industrial applications. In cases where superior performance through homozygosity can be narrowed down to specific heteroalleles, it may be possible to use targeted allele engineering approaches for strain improvement, minimizing or eliminating the negative effects of losing heterozygosity in the neighboring loci. In addition, it would be interesting to characterize these strain sets under conditions designed to more closely recapitulate the bioethanol fermentation environment (Raghavendran et al., 2017), as well as in actual industrial scale sugarcane distilleries. Those experiments would be extremely valuable to determine whether any of the inbred or UPD strains are capable of outperforming JAY270 when challenged by multiple simultaneous stressors. Finally, our work demonstrated the use of the heterologous *AmdS* counter-selectable marker for induction of targeted chromosome loss, which allowed us to bypass the need to introduce auxotrophic markers that often influence yeast phenotypic analyses (Swinnen et al., 2015), while also broadening the UPD approach applicability to wild and industrial strains.

## Material and Methods

### Growth Media

Yeast cells were grown in YPD (20 g/L glucose, 20 g/L peptone, 10 g/L yeast extract, 20 g/L bacteriological agar for solid media), unless otherwise noted. Transformants carrying the *GFP-kanMX* cassette were selected in YPD plates supplemented with 400 mg/L of geneticin. Selection of *AmdS* positive (*Amds*+) clones was performed in acetamide media (20 g/L glucose, 6.6 g/L potassium sulfate, 1.7 g/L YNB without amino acids, 0.6 g/L acetamide, 20 g/L bacteriological agar). Fluoroacetamide media was used for *AmdS* counter-selection (20 g/L glucose, 5 g/L ammonium sulfate, 1.7 g/L YNB without amino acids, 1.4 g/L complete drop-out mix, 2.3 g/L fluoroacetamide, 20 g/L bacteriological agar). Spot assays for phenotypic screening of the inbred collection was performed in different types of media, including: 2% YPGE (20 g/L peptone, 10 g/L yeast extract, 30 mL/L glycerol; 30 mL/L 100% ethanol, 20 g/L bacteriological agar), 2% YP Galactose (20 g/L galactose, 20 g/L peptone, 10 g/L yeast extract, 20 g/L bacteriological agar), 2% YP Raffinose (20 g/L raffinose, 20 g/L peptone, 10 g/L yeast extract, 20 g/L bacteriological agar).

### Yeast Genetic Backgrounds and Microbiology Procedures

All *S. cerevisiae* strains used in this study descended from the JAY270 background (**Table S1**) (Argueso et al., 2009). Standard procedures for yeast culture, transformation, crossing and sporulation were followed (Ausubel et al., 2003).

#### Construction of a Collection of Inbred Diploids Derived From JAY270

A detailed description of the strategy adopted to construct the inbred strain collection is described in Results section. Genome sequencing data associated with this study is available in the Sequence Read Archive (SRA) database under study number SRP082524 and described previously (Rodrigues-Prause et al., 2018).

#### Construction of a GFP-Tagged JAY270 Derivative

A *GFP-KanMX* cassette with homology to a non-coding region located 365 bp upstream to the centromere 5 (*CEN5*, genomic coordinate = 151,522) was built. A *GFP* cassette was amplified from pFA6a-TEF2P-GFP-ADH1-NATMX4, kindly provided by Dr. Maitreya Dunham’s laboratory, using the primers JAO1385 and JAO1386. The *KanMX4* cassette was amplified from pFA6-KanMX4 using primers JAO1387 and JAO466 (Wach et al., 1994). Both cassettes were fused by double-joining PCR and transformed into JAY270. Four transformants were selected, purified and tested in 22-cycles of co-culture with the wild type JAY270 strain, one of which (JAY2208) was used for the co-culture competitions against the inbred and UPD strains. See **Table S2** for the primers used in this construction.

#### Construction of UPD Strains

Destabilization of centromere function was achieved by the insertion of the counter-selectable gene *AmdS* (Solis-Escalante et al., 2013) at the consensus centromeric region of each chromosome analyzed. Cassettes targeting different integration sites (∼100 bp or ∼5 bp upstream to the targeted centromere), as well as including or excluding a terminator sequence, were tested. Clones showing UPD were more frequently recovered when cassettes that excluded the terminator region of *AmdS* were integrated immediately upstream to the consensus centromere sequence (data not shown). All cassettes were amplified from pCfB2399 (Stovicek et al., 2015) (gift from Irina Borodina; addgene plasmid # 67550) and targeted *CEN4*, *CEN14* and *CEN15*. Transformants were selected and purified in acetamide media. The integration of the cassettes was confirmed by PCR that amplified the left and right junctions between the *AmdS* cassette and the centromeric region. PCR products were designed to span at least one centromere-proximal HetSNP that was used to determine in which homolog the integration occurred using Sanger sequencing. At least two independent clones containing the insertion in each homolog were selected. Cells were grown on YPD plates for 24 h to allow for loss of the *AmdS*-marked homolog, diluted in 200 µl of water and plated in the counter-selection fluoroacetamide media. At least four types of cells should be able to grow in this selective media: (1) cells with inactivation of the *AmdS* gene through point mutation; (2) cells that acquired loss-of-heterozygosity (LOH) tracts spanning *AmdS* as a result of a recombination event, such as gene conversion or mitotic crossover leading to LOH, (3) cells that lost the whole homolog containing *AmdS* and persisted as monosomics; and (4), our targeted UDP class, cells that lost the whole homolog containing *AmdS*, undergoing a transient monosomic state followed by endoduplication of the remaining homolog. Two sequential tests were performed to screen for targeted UDP clones. First, candidates were genotyped using PCR restriction fragment length polymorphism (PCR-RFLP; **Table S5**) analysis at two centromere-distal positions (HetSNPs near the left and right ends of the chromosome arms, and near the centromeric region). Clones genotyped as homozygous at all three markers could be either monosomic or homozygous disomic for the chromosome of interest. To distinguish between these cell types, candidates were sporulated and tetrads were dissected. Monosomic clones should generate tetrads with two viable and two inviable spores, whereas UPD clones should generate tetrads with four viable spores. Candidates that were homozygous for all three PCR-RFLP markers and produced tetrads with four viable spores were selected for phenotypic tests. See **Table S2** for the primers used in the construction and validation of these UPD strains.

### Phenotypic Assessment of the Inbred Collection

#### Phenotypic Screenings Through Plate Spotting Assay

Three cultures of JAY270 and of each inbred strain were grown to saturation at 30°C in 96-well plates containing 200 µl of YPD. Cultures were diluted by immersing a 96-pin replicator in the resuspended saturated cultures and subsequently in a 96-well plate containing 100 µL of distilled water. Diluted cells were pinned in different types of plates and allowed to grow under different conditions as detailed in **Table S3**.

#### Heat Stress Tolerance Assay Using Colony Size Scores

Cells were thawed from −80°C freezer stocks and incubated at 30°C in YPD plates for 24 h. Cells were inoculated into 5 ml liquid YPD, and incubated for 24 h at 30°C in a rotating drum. Saturated cultures were diluted 10,000-fold and 40 µL were plated onto four YPD plates, two of which were incubated for 48 h at 30°C and two were incubated for 96 h at 39°C. Growth at high temperature between strains was assessed through a colony size scoring system (**Figure S4**). Assays were repeated independently at least three times for the whole collection of inbred strains. Median scores between repetitions were calculated for each strain, and standard error of the median was determined by multiplying the standard error of the mean by 1.253 (or square root of π/2).

#### Heat Stress Tolerance Assay in Liquid Cultures

Cells were thawed from −80°C freezer stocks and incubated at 30°C on YPD plates for 48 h. Cells were inoculated into 5 ml liquid YDP and incubated for 24 h in a cell culture-rotating drum at 30°C. Saturated cultures were diluted 10-fold and 10 µL were used to inoculate 5 ml of liquid YPD. Cultures were incubated for 24 h in a rotating drum in a warm room at 39°C. OD600 was determined using a spectrophotometer at 0, 12, 22, and 24 h of incubation. To assure cultures incubated at 39°C started with a comparable number of cells, the saturated cultures used as inoculum were also plated on YPD (50 µL, 10,000-fold dilution) and colony-forming units (CFU/mL) were determined after 48 h incubation at 30°C (data not shown). This assay was repeated independently two times for the collection of UPD strains with three biological replicates per strain each time.

### Flow Cytometry-Based Competitive Growth Fitness Assay

Yeast cells were thawed from −80°C freezer stocks and incubated at 30°C on YPD plates for 24 h. Cells were inoculated into 5 ml liquid YPD, and grown until saturation for 24 h at 30°C in a rotating drum. Equal volumes of each inbred culture and the JAY270-GFP marked culture were mixed and used to inoculate three assay tubes containing 5 ml of fresh liquid YPD, establishing “Cycle 0” of the competition assay. An aliquot of each mixture was also run through the flow cytometer to determine the starting (pre-culture) percentages of inbred (GFP−) and JAY270-GFP marked cells. Co-cultures were incubated at 30°C in a rotating drum, and every 24 h (one cycle of competition) 1% of the co-culture volume (50 µL) was transferred to 5 mL of fresh YPD medium. Each co-culture competition was performed in triplicate. The percentages of inbred and JAY270 cells was assessed at the beginning of cycle 0 and at the end of cycles 2, 5 and 8 using a Cyan ADP7 color flow cytometer coupled to a HyperCyt Rapid Sampler system for 96-well plate-based assays. Ninety-six well plates for flow cytometry readings were prepared by diluting 10 µl of each culture in 190 µl of 1% PBS buffer. A PBS-only well was placed after each triplicate and triplicates of a control competition between unmarked JAY270 and JAY270–GFP were included every time a new experiment was initiated. Flow cytometry parameters were optimized by applying a series of gatings that excluded from the analysis cell debris (**Figure S5A**) and cell agglomerates (**Figure S5B**), resulting in a final cell count that was gated into FITC− and FITC+ populations based on their fluorescence signals (**Figure S5C**).

### Genome-Wide Association Analyses in the Inbred Collection

#### Genotype Calling of Inbred Diploids

A previously described phased map of 12,023 heterozygous SNPs (HetSNPs) in JAY270’s genome (**File S1**) (Rodrigues-Prause et al., 2018) was used for calling the genotype of the recombinant haploid strains that originated the collection of inbreds. The phased JAY270 HetSNP haplotypes were arbitrarily designated as maternal (M) or paternal (P).

CLC genomics workbench software was used for mapping sequencing reads from each parental recombinant haploid onto the S288c reference and detecting SNPs across their whole genome. The nucleotides present at each of the 12,023 loci in the JAY270 HetSNP list were determined for each haploid. When no SNPs were detected at those positions, the reference nucleotide genotype was called, while the alternative nucleotide was called when the alternative SNP was detected at a frequency higher than 0.95. After the genotypes were determined they were converted to the respective haplotype designations as M or P.

In order to deduce the diploid genotype of each partial inbred strain, we examined the genotype of their respective parents for each of the 12,023 HetSNPs. Heterozygous M/P loci were called whenever the haploid parents presented distinct nucleotides at a specific position. Whenever the parents presented the same nucleotide at a specific position, the locus was designated either homozygous M/M or homozygous P/P. A genotype map of all haploid parents and inbred strains is provided in **File S1**.

#### Statistical Analysis of Genotype/Phenotype Association

Analysis was done using R version 3.4.0, the R/qtl package version 1.42-8, SAS version 9.4, and the SAS GLM procedure (Broman et al., 2003; SAS, 2013; R_Core_Team, 2017). Phenotype data from two independent assays (high temperature stress tolerance score and percent inbred cells in co-culture competition at cycle 5) and genotypes at 11,742 HetSNPs across the genome from 78 partial inbred strains were used as inputs for the QTL analysis (Results section, **File S1**, and **Table S4**). Each phenotype was analyzed separately using a one-dimensional scan of the genome using Haley-Knott regression, and log10 likelihood ratio (LOD) scores were determined for each marker position. Five independent 10,000 iteration permutation tests were run to determine the null distribution of our data and the genome-wide LOD threshold value (median of five permutations). Using the 95^th^ percentile of the distribution of maximum LOD scores generated from the permutation tests, this resulted in genome-wide LOD thresholds of LOD > 4.11 for heat stress tolerance and LOD > 4.11 for co-culture competition. To determine the allele (M or P) contributing the higher phenotypic value at each locus, we simply noted which genotype showed the highest mean phenotype value. We used two methods to determine the inheritance model of each QTL (additive, dominant/recessive, or overdominance), focusing on the significant regions in the genome and the marker with the highest LOD score for each region. First, each significant region of the genome was visually inspected using effect plots to show the mean phenotype values for each genotype at each locus of interest. Second, Tukey pairwise comparisons were used to determine which genotypes had significantly different mean phenotype values from each other at an alpha level of 0.05 at each locus of interest. Plots showing magnitude of LOD value vs. whole genome position were generated using the ggplot2 package version 2.2.1 using a custom script (Wickham, 2009). Three statistically significant regions were excluded from further analysis and subsequently not color-coded in **Figure 4** due to having no ORFs in the region (Competitive growth: Chr5, Heat Stress: Chr7, Chr14).

A two-dimensional scan of the genome was also performed, but no evidence was found for interactions between QTL (data not shown).

Loci identified through the one-dimensional scans and the genome-wide LOD threshold using Haley-Knott regression (R/qtl package) were interrogated further to determine the percent variance explained (PVE) by each QTL using the SAS GLM (general linear models) procedure. Single-factor ANOVA was used to assess the association between genotype frequencies at individual loci and each phenotype (high temperature stress tolerance and co-culture competitive growth), using a comparison-wise error rate of 0.05. Least squares means of genotypic classes were calculated with the LSMEANS option of the GLM procedure. PVE was determined by multiplying the R^2 value in the ANOVA output by 100, and p-values were obtained. This procedure can result in relatively high PVE values for each locus that totaled to over 100%, thus overestimating the absolute single locus PVE. Thus, we also calculated relative contribution of the loci to serve as a comparison parameter. In a second approach to estimate PVEs, multi-locus models were fit for each phenotype and tested with the GLM procedure. The model presented in **Table S8** explained the highest proportion of the phenotypic variance while, at the same time, individual loci were significant at the 0.01 significance level.

## Author Contributions

NS and JA conceived the study. NS, RW, and JA designed and performed the experiments, analyzed data, generated figures, and wrote the manuscript.

## Funding

NS received a pre-doctoral fellowship from Brazil’s CAPES (0316/13-0). Research reported here was supported by an NIH grant to JA (R35GM119788).

## Conflict of Interest Statement

The authors declare that the research was conducted in the absence of any commercial or financial relationships that could be construed as a potential conflict of interest.
