# Supplementary material for: Controlled Reduction of Genomic Heterozygosity in an Industrial Yeast Strain Reveals Wide Cryptic Phenotypic Variation
Source: Front Genet. 2019 Sep 11;10:782. doi: 10.3389/fgene.2019.00782 (PMC6749062; doi:10.3389/fgene.2019.00782)
Supplement: Supplementary file 1 [file DataSheet_1.pdf]

Figure S1, Sampaio *et al.*

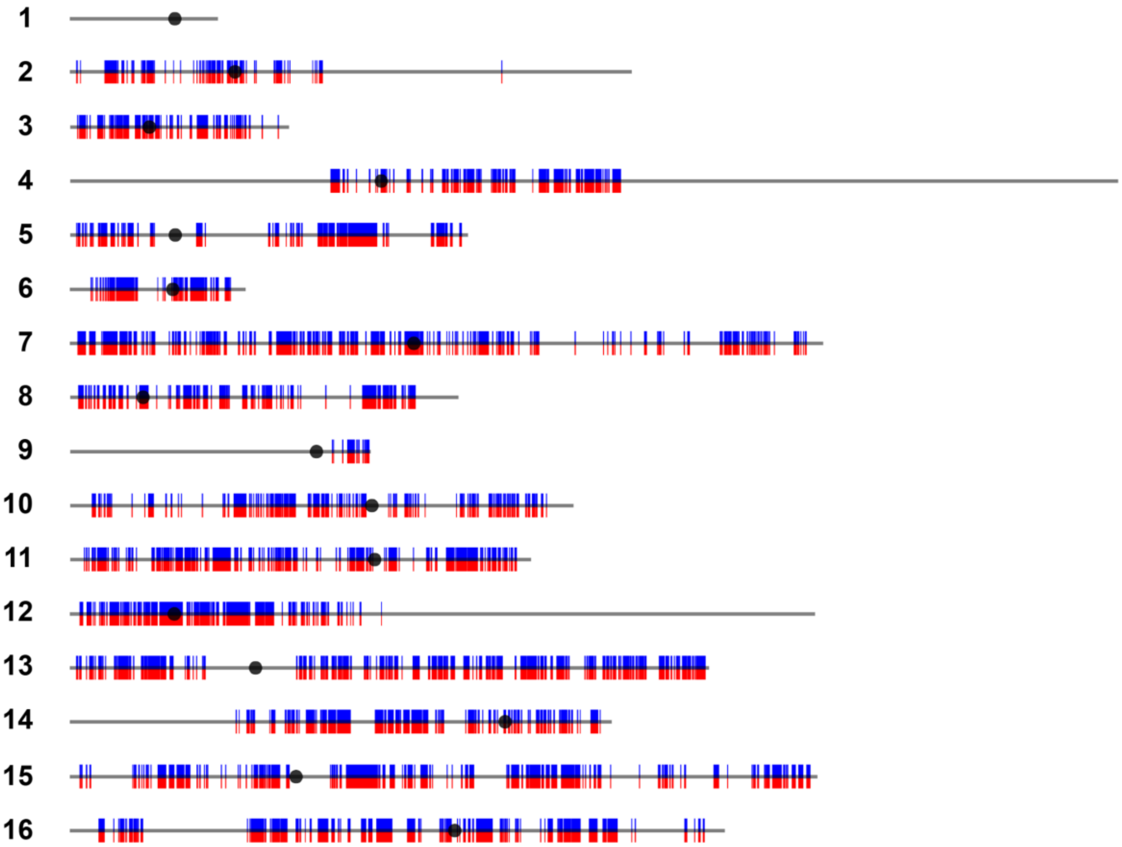

Figure S2, Sampaio *et al.*

A.

MATα-ACE2 **Haploids** (columns) × ***MATa-ace2-A7*** **Haploids** (rows)

	JAY#	*T1*	*T2*	*T3*	*T4*	*T5*	*T6*	*T7*	*T8*	*T9*	*T10*	*T11*	*T12*	*T13*
		1520	***1525***	***1526***	***1531***	***1536***	***1539***	***1545***	***1546***	***1553***	***1557***	***290***	***293***	***328***
T1	***1521***		1598	1600	1602	1604	1606	1608	1610	1612	1614	1616	1618	1620
T2	***1522***			1622	1624	1626	1628	1630	1632	1634	1636	1638	1640	1642
T3	***1529***				1644	1646	1648	1650	1652	1654	1656	1658	1660	1662
T4	***1533***					1664	1666	1668	1670	1672	1674	1676	1678	1680
T5	***1535***						1682	1684	1686	1688	1690	1692	1694	1696
T6	***1540***							1698	1700	1702	1704	1706	1708	1710
T7	***1543***								1712	1714	1716	1718	1720	1722
T8	***1549***									1724	1726	1728	1730	1732
T9	***1552***										1734	1736	1738	1740
T10	***1554***											1742	1744	1746
T11	***291***												1748	1750
T12	***295***													1752
T13	***326***													

B.

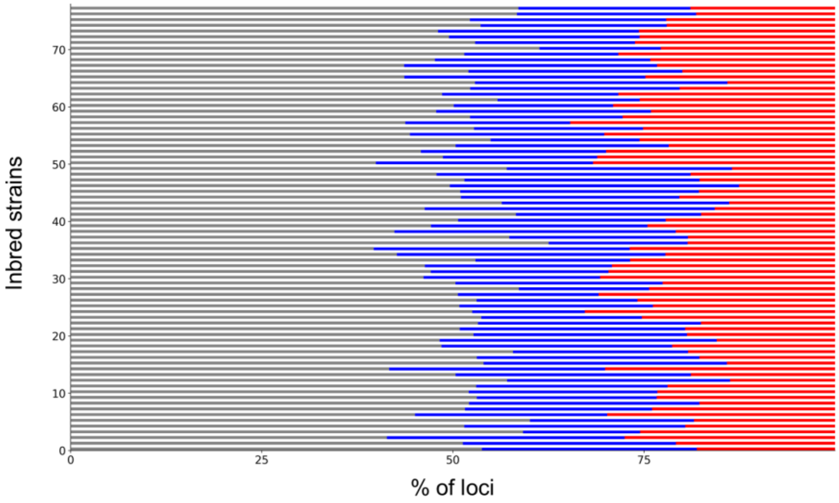

C.

78 strains in inbred collection	Genotype Frequency (%)		
	M/P	P/P	M/M
Average	51.02	26.11	22.87
Median	51.49	25.98	22.68
Maximum	62.52	37.95	34.65
Minimum	39.64	14.72	12.57

Figure S3, Sampaio *et al.*

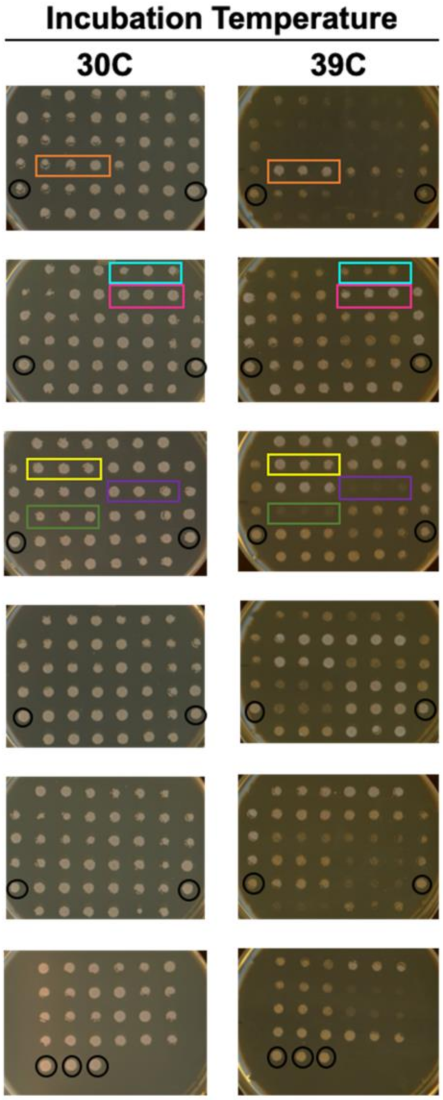

Figure S4, Sampaio *et al.*

**Phenotype classification**	**Representative growth at 39C**	**Representative growth at 30C**
**Score 1** (JAY1664)<br><br>Very small colonies, at the lower limit of unaided visualization.	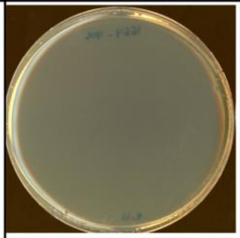	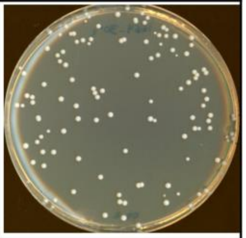
**Score 2** (JAY1628)<br><br>Small, yet easily visible, colonies.	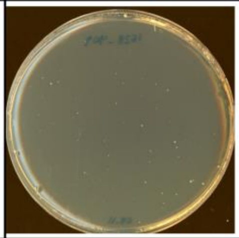	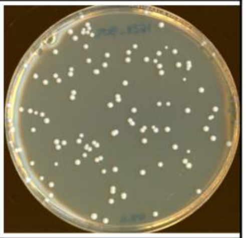
**Score 3** (JAY270)<br><br>Heterogeneous colony size, ranging from medium to small sizes.	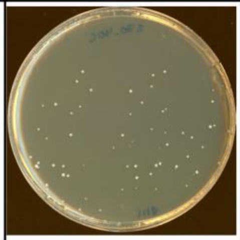	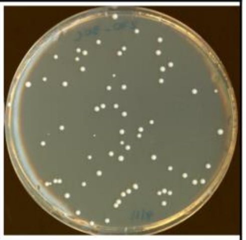
**Score 4** (JAY1658)<br><br>Medium to large colonies, with most having medium size. Narrow variation in colony size.	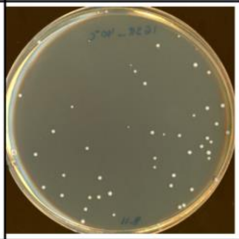	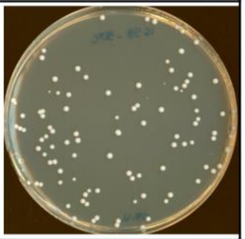
**Score 5** (JAY1610)<br><br>Large colonies. Homogenous size.	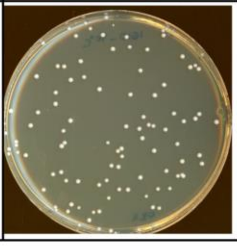	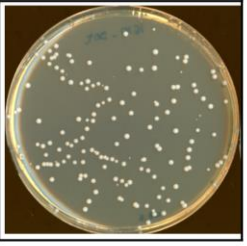

Figure S5, Sampaio *et al.*

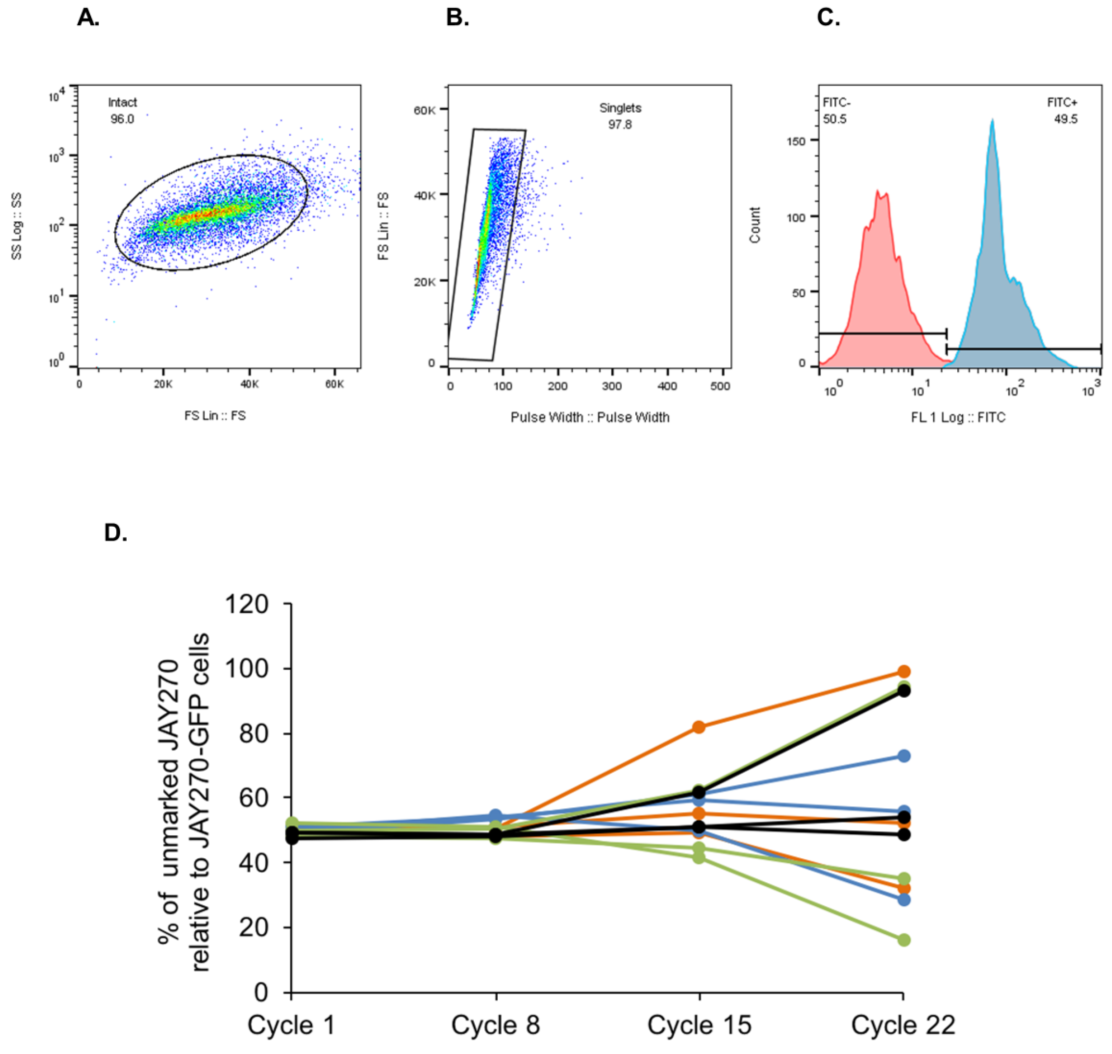

Figure S6, Sampaio *et al.*

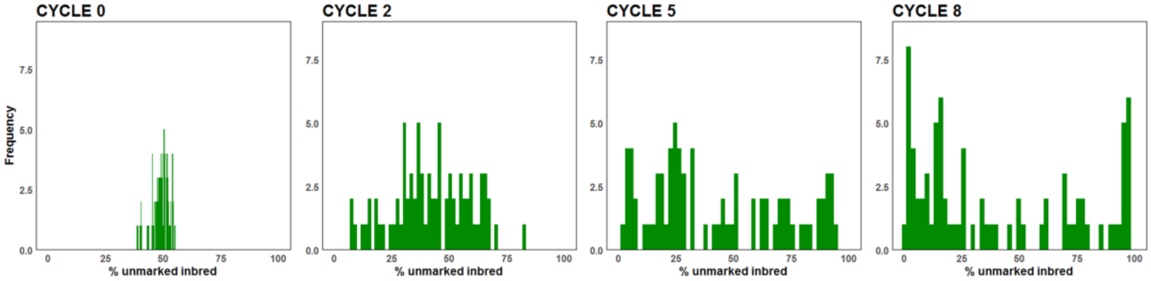

Figure S7, Sampaio *et al.*

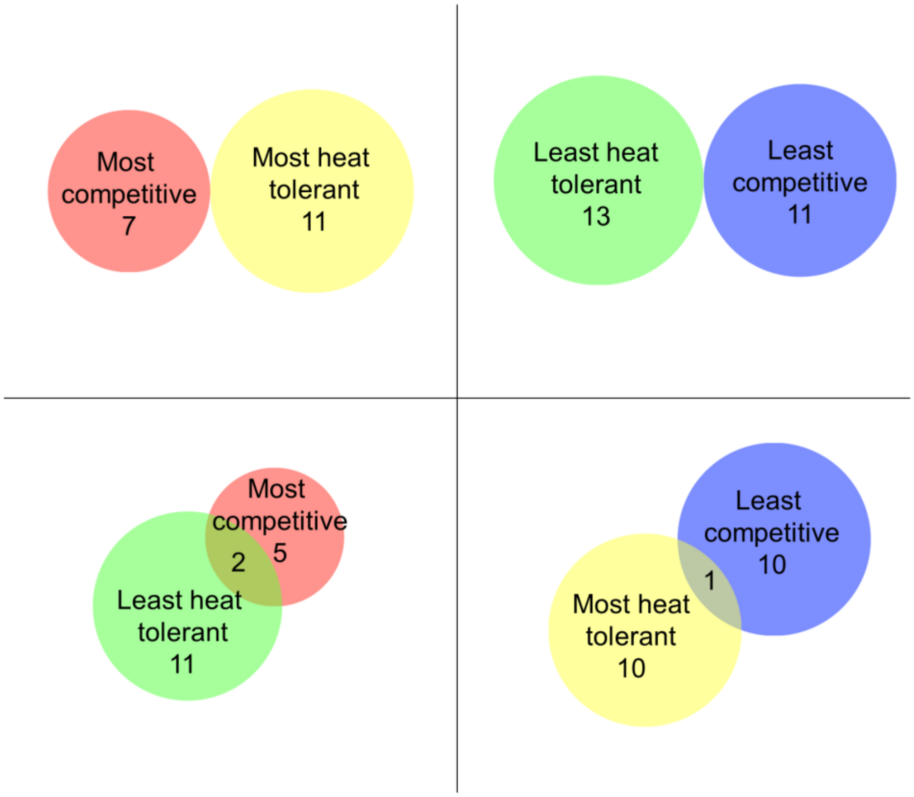

Figure S8, Sampaio *et al.*

A.

Chromosome	Fluoroacetamide resistant clones with LOH of both distal markers / total clones screened by PCR-RFLP	
	AmdS near M centromere	*AmdS* near P centromere
Chr4	4 / 20 (20.0%)	8 / 82 (9.8%)
Chr14	12 / 12 (100%)	11 / 12 (91.7%)
Chr15	8 / 16 (50.0%)	11 / 42 (26.2%)

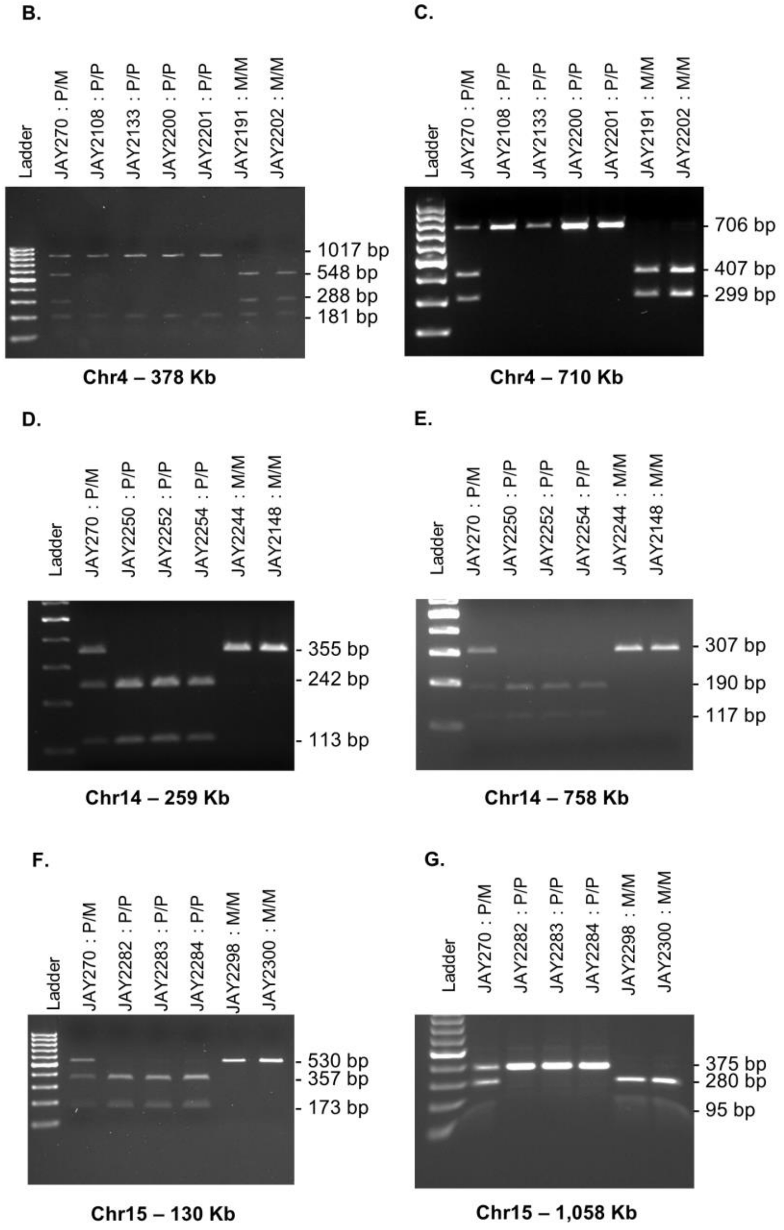

Figure S9, Sampaio *et al.*

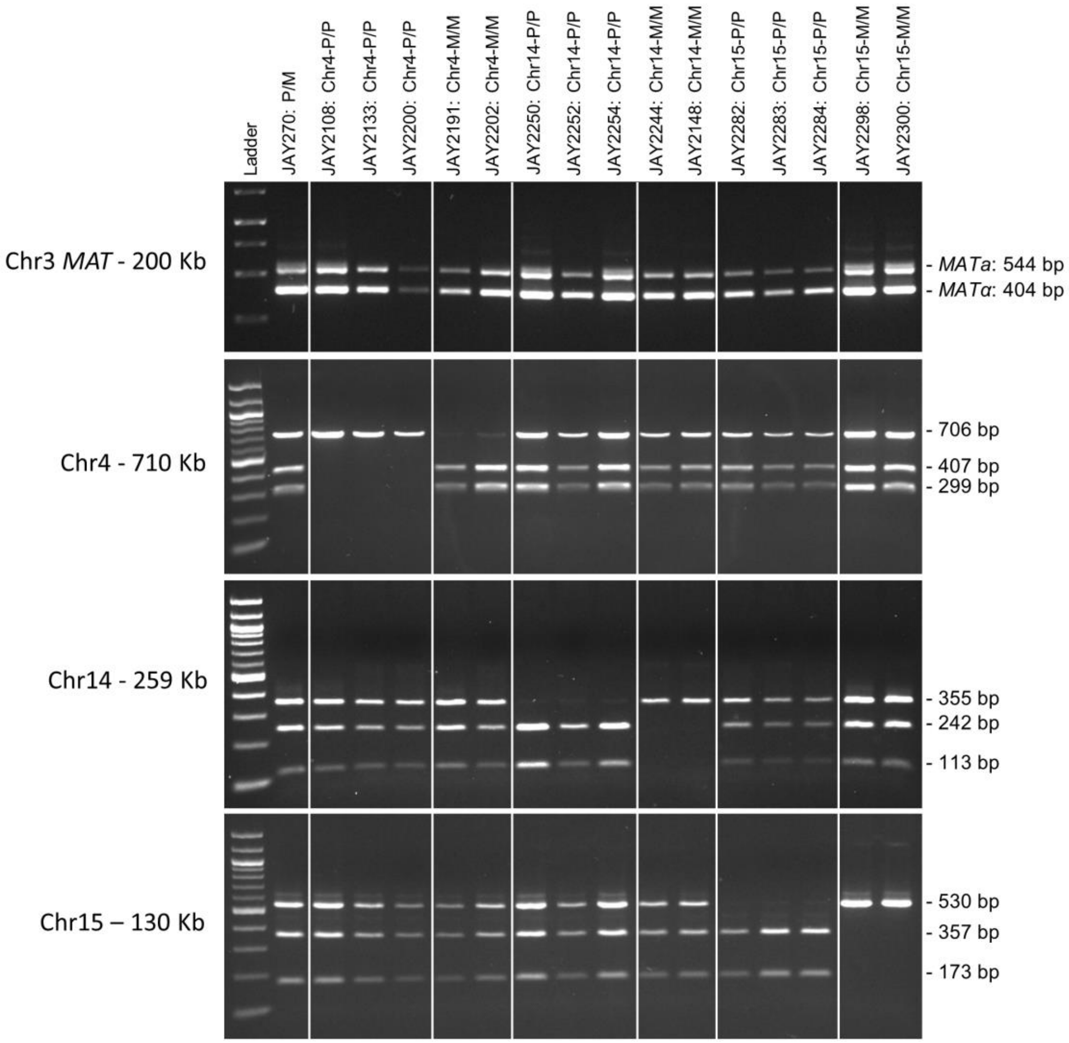

Figure S10, Sampaio *et al.*

A. Colony formation in agar media at 39C heat tolerance scores

Strain	Description	Heat tolerance phenotype scores (based on colony size)					
		Consensus score	Independent biological replicates				
			A	B	C	D	E
**JAY1632**	Heat tolerant inbred	5	4	5	5	5	4
**JAY1610**	Heat tolerant inbred	5	5	4	5	5	4
**JAY270**	Parental strain	3	3	2	3	–	–
**JAY1666**	Heat sensitive inbred	1	2	2	1	1	1
**JAY1664**	Heat sensitive inbred	1	1	1	1	1	1

B. Liquid media 39C growth curves

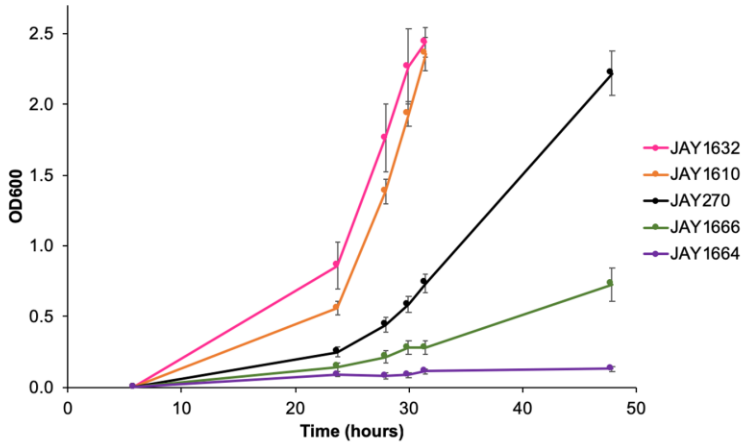

Supplemental Figures, Sampaio *et al*.

Figure S1. Draft map of heterozygosity in the JAY270 genome (Rodrigues-Prause et al., 2018), showing the distribution of 12,023 phased heterozygous single nucleotide polymorphisms (HetSNPs; File S1; double colored markers, arbitrarily defined as maternal (red) or paternal (blue) haplotypes). Each horizontal line represents a *S. cerevisiae* chromosome. Black circles indicate the positions of the centromeres. Plots were generated to scale in Python 2.7 using the matplotlib package and a custom script. For size reference, Chr1 is 230 Kb. Note that Chr1 is fully homozygous.

Figure S2. Inter-tetrad matings and genotype distribution in the inbred collection.
A) Schematic representation of the matings between recombinant haploids. Two haploid spores from each of 13 different tetrads (T1 – T13) were selected to generate a collection of diploid inbred strains resulting from inter-tetrad matings. The JAY number (*e.g.*, JAY1520, JAY1598, etc.) corresponding to the strain identifier is displayed for the parent haploids and for the inbreds, and matches the entries in Table S1.
B) The inbreds were expected to contain approximately 50% of the original heterozygosity present in the parental JAY270 genome, and be homozygous for 25% of the paternal alleles and 25% of the maternal alleles. The genotype classes in the inbred collection from panel A and Figure 1B are plotted as cumulative frequencies for all HetSNP markers. Heterozygous M/P (gray); homozygous P/P (blue); homozygous M/M (red). C) Summary of the frequency of genotype classes in the inbred collection.

Figure S3. Variation in tolerance to heat stress in the inbred collection. A plate spotting assay was used for phenotypic screening. The column to the left shows uniform growth among strains incubated for 24 hours at 30C. The column to the right shows variable growth at 39C after four days of incubation. Black circles highlight the control JAY270, which displays an intermediate level of heat tolerance. Rectangles highlight the growth of representative strains shown in Figures S4 and S8 with different levels of heat tolerance, from high to low: JAY1632 (pink), JAY1610 (orange), JAY1658 (yellow), JAY1628 (turquoise), JAY1666 (green), and JAY1664 (purple).

Figure S4. Inbred strains show phenotypic variation in tolerance to heat stress. A scoring system was used to assess the heat stress tolerance phenotype of each inbred strain. The criteria for each score used and the strains chosen as representative examples for each phenotypic category are described next to images of growth on Petri dishes. Incubation at 39C for 4 days and incubations at 30C for 2 days are depicted.

Figure S5. Flow cytometry analysis.
A) False events corresponding to cell debris or other material were eliminated by gating intact cells in a side scatter by forward scatter plot.

B) Potential cell aggregates were subsequently removed from counting by gating single cells in a forward scatter by pulse width plot.
C) Final counts of GFP+ (JAY270 parent strain) and GFP- cells (inbred diploid strains) in each co-culture were determine by gating the FITC+ and FITC- population in a count by FITC signal plot.
D) Co-culture competition assays between four independently-generated GFP-marked JAY270 derivatives and the unmarked JAY270 parent over 22 passage cycles (24 hours per cycle). Each of the four GFP-marked controls (shown in orange, blue, green and black curves) were co-cultured with the original JAY270 parent in triplicates. A neutral and stable ratio of GFP- to GFP-tagged strains was observed in all cases through cycle 8 of competition. Beyond that point the ratios tended to diverge up or down between triplicates, presumably as a result of emergence of beneficial *de novo* mutations either in the GFP- or GFP+ competitors. These results indicated that the GFP marker itself did not confer an advantage or a burden to the cells carrying it, and that any departure from the starting 1:1 ratio detected before cycle 8 could be confidently attributed to the genotypes present in the starting inoculum, and not due to *de novo* mutations arising during the co-culture.

Figure S6. Distributions of the competitive growth phenotypes in the inbred strain collection in cumulative co-culture over time. Histograms showing the change over time of the distributions of phenotypes in the competitive growth assay. By Cycle 8 the cumulative nature of this phenotype can be seen by the higher numbers of strains in the highest and lowest phenotypes.

Figure S7. Venn diagrams displaying pairwise overlap comparisons of inbred strains that displayed extreme phenotypes in heat tolerance and competitive growth (at Cycle 5). Circles represent the groups of inbred strains that were most heat tolerant (yellow) and most competitive (red); and the groups that were least heat tolerant (green) and least competitive (purple). Diagrams were created using BioVenn web application (Hulsen et al., 2008)

Figure S8. Validation of targeted UPD strain constructions.
A) Table showing the frequency of concomitant loss of both (left and right arm) distal PCR-RFLP markers physically linked to the insertion of *AmdS* adjacent to the respective centromere (Figure 5A). A subset of the *AmdS*$^-$ clones remained heterozygosis at one or both distal PCR-RFLP markers, suggesting that they were likely derived from mitotic recombination instead of the desired chromosome loss. All of the chromosome loss clones with bilateral LOH analyzed by tetrad dissection displayed full (4) spore viability, indicating that spontaneous endoduplication leading to UPD occurred very quickly after selection of the monosomic intermediates.
B-G) Agarose gel electrophoresis of PCR-RFLP genotyping of HetSNP markers. Panels B, D and F show genotypes of RFLP markers located at the left arms of Chr4, Chr14 and Chr15, respectively. Panels C, E and G show genotypes of RFLP markers located at the right arms of Chr4, Chr14 and Chr15, respectively. The approximate coordinates of each RFLP marker are indicated below each image. The approximate positions of the relevant centromeres where *AmdS* was inserted are: *CEN4* – 450 Kb; *CEN14* – 629 Kb; *CEN15* – 327 Kb. The

corresponding genotype at each position is indicated for each UPD strain as P/P for homozygous paternal, M/M for homozygous maternal and P/M for heterozygous.

Figure S9. Validation of retention of heterozygosity at non-targeted loci in the UPD strains. PCR-RFLP genotyping of three non-targeted and one targeted HetSNP markers was performed for JAY270 and each of the 15 independently generated UPD strains constructed for this study. Panels show agarose gels used for genotyping of PCR-RFLP markers located at the Chr3 *MAT* locus (not targeted in any of the UPD strains), and at each of the targeted chromosomes. Note that all UPD strains remained heterozygous at the non-targeted chromosomes but were homozygous for the specific allele targeted. Vertical lines were used to facilitate the visualization of the groups of strains with the same targeted UPDs.

Figure S10. Comparison of the two assays used to characterize heat stress tolerance.
A) Representative results from colony size (agar media; 4 days incubation) scoring system described in Figure S4 and used in Figure 3A.
B) Pure culture (liquid media) growth curves and OD600 measurements over a period of 24-48 hours. Error bars shown in SD.
Strains used in the two heat tolerance assays are color coded similarly in A and B and Figure S3.

Table S1. Yeast strains used in this study.

Strain	Mating type	Relevant genotype	Description	Source
**Haploids:**				
JAY1520	*MATα*	*ACE2*	Sibling spores from a JAY270 tetrad	(Rodrigues-Prause et al., 2018)
JAY1521	*MATa*	*ace2-A7*		
JAY1525	*MATα*	*ACE2*	Sibling spores from a JAY270 tetrad	(Rodrigues-Prause et al., 2018)
JAY1522	*MATa*	*ace2-A7*		
JAY1526	*MATα*	*ACE2*	Sibling spores from a JAY270 tetrad	(Rodrigues-Prause et al., 2018)
JAY1529	*MATa*	*ace2-A7*		
JAY1531	*MATα*	*ACE2*	Sibling spores from a JAY270 tetrad	(Rodrigues-Prause et al., 2018)
JAY1533	*MATa*	*ace2-A7*		
JAY1536	*MATα*	*ACE2*	Sibling spores from a JAY270 tetrad	(Rodrigues-Prause et al., 2018)
JAY1535	*MATa*	*ace2-A7*		
JAY1539	*MATα*	*ACE2*	Sibling spores from a JAY270 tetrad	(Rodrigues-Prause et al., 2018)
JAY1540	*MATa*	*ace2-A7*		
JAY1545	*MATα*	*ACE2*	Sibling spores from a JAY270 tetrad	(Rodrigues-Prause et al., 2018)
JAY1543	*MATa*	*ace2-A7*		
JAY1546	*MATα*	*ACE2*	Sibling spores from a JAY270 tetrad	(Rodrigues-Prause et al., 2018)
JAY1549	*MATa*	*ace2-A7*		
JAY1553	*MATα*	*ACE2*	Sibling spores from a JAY270 tetrad	(Rodrigues-Prause et al., 2018)
JAY1552	*MATa*	*ace2-A7*		
JAY1557	*MATα*	*ACE2*	Sibling spores from a JAY270 tetrad	(Rodrigues-Prause et al., 2018)
JAY1554	*MATa*	*ace2-A7*		
JAY290	*MATα*	*ACE2*	Sibling spores from a JAY270 tetrad	Our strain collection
JAY291	*MATa*	*ace2-A7*		
JAY293	*MATα*	*ACE2*	Sibling spores from a JAY270 tetrad	Our strain collection
JAY295	*MATa*	*ace2-A7*		
JAY328	*MATα*	*ACE2*	Sibling spores from a JAY270 tetrad	Our strain collection
JAY326	*MATa*	*ace2-A7*		
**Diploids:**				
JAY270	*MATα/MATa*	*ACE2/ace2-A7*	Sugarcane bioethanol fermentation strain	Argueso et al., 2009
JAY2208 (JAY270-GFP)	*MATα/MATa*	*ACE2/ace2-A7; CEN5::GFP-KanMX*	Hemizygous *GFP-KanMX* insertion near Chr5	This study
JAY1598	*MATα/MATa*	*ACE2/ace2-A7*	Inbred diploid resulting from crossing between JAY1521 x JAY1525	This study
JAY1600	*MATα/MATa*	*ACE2/ace2-A7*	Inbred diploid resulting from crossing between JAY1521 x JAY1526	This study
JAY1602	*MATα/MATa*	*ACE2/ace2-A7*	Inbred diploid resulting from crossing between JAY1521 x JAY1531	This study
JAY1604	*MATα/MATa*	*ACE2/ace2-A7*	Inbred diploid resulting from crossing between JAY1521 x JAY1536	This study
JAY1606	*MATα/MATa*	*ACE2/ace2-A7*	Inbred diploid resulting from crossing between JAY1521 x JAY1539	This study
JAY1608	*MATα/MATa*	*ACE2/ace2-A7*	Inbred diploid resulting from crossing between JAY1521 x JAY1545	This study
JAY1610	*MATα/MATa*	*ACE2/ace2-A7*	Inbred diploid resulting from crossing between JAY1521 x JAY1546	This study
JAY1612	*MATα/MATa*	*ACE2/ace2-A7*	Inbred diploid resulting from crossing between JAY1521 x JAY1553	This study
JAY1614	*MATα/MATa*	*ACE2/ace2-A7*	Inbred diploid resulting from crossing between JAY1521 x JAY1557	This study
JAY1616	*MATα/MATa*	*ACE2/ace2-A7*	Inbred diploid resulting from crossing between JAY1521 x JAY290	This study
JAY1618	*MATα/MATa*	*ACE2/ace2-A7*	Inbred diploid resulting from crossing between JAY1521 x JAY293	This study

Table S1 (continued). Yeast strains used in this study.

Strain	Mating type	Relevant genotype	Description	Source
JAY1620	*MATα/MATa*	*ACE2/ace2-A7*	Inbred diploid resulting from crossing between JAY1521 x JAY328	This study
JAY1622	*MATα/MATa*	*ACE2/ace2-A7*	Inbred diploid resulting from crossing between JAY1522 x JAY1526	This study
JAY1624	*MATα/MATa*	*ACE2/ace2-A7*	Inbred diploid resulting from crossing between JAY1522 x JAY1531	This study
JAY1626	*MATα/MATa*	*ACE2/ace2-A7*	Inbred diploid resulting from crossing between JAY1522 x JAY1536	This study
JAY1628	*MATα/MATa*	*ACE2/ace2-A7*	Inbred diploid resulting from crossing between JAY1522 x JAY1539	This study
JAY1630	*MATα/MATa*	*ACE2/ace2-A7*	Inbred diploid resulting from crossing between JAY1522 x JAY1545	This study
JAY1632	*MATα/MATa*	*ACE2/ace2-A7*	Inbred diploid resulting from crossing between JAY1522 x JAY1546	This study
JAY1634	*MATα/MATa*	*ACE2/ace2-A7*	Inbred diploid resulting from crossing between JAY1522 x JAY1553	This study
JAY1636	*MATα/MATa*	*ACE2/ace2-A7*	Inbred diploid resulting from crossing between JAY1522 x JAY1557	This study
JAY1638	*MATα/MATa*	*ACE2/ace2-A7*	Inbred diploid resulting from crossing between JAY1522 x JAY290	This study
JAY1640	*MATα/MATa*	*ACE2/ace2-A7*	Inbred diploid resulting from crossing between JAY1522 x JAY293	This study
JAY1642	*MATα/MATa*	*ACE2/ace2-A7*	Inbred diploid resulting from crossing between JAY1522 x JAY328	This study
JAY1644	*MATα/MATa*	*ACE2/ace2-A7*	Inbred diploid resulting from crossing between JAY1529 x JAY1531	This study
JAY1646	*MATα/MATa*	*ACE2/ace2-A7*	Inbred diploid resulting from crossing between JAY1529 x JAY1536	This study
JAY1648	*MATα/MATa*	*ACE2/ace2-A7*	Inbred diploid resulting from crossing between JAY1529 x JAY1539	This study
JAY1650	*MATα/MATa*	*ACE2/ace2-A7*	Inbred diploid resulting from crossing between JAY1529 x JAY1545	This study
JAY1652	*MATα/MATa*	*ACE2/ace2-A7*	Inbred diploid resulting from crossing between JAY1529 x JAY1546	This study
JAY1654	*MATα/MATa*	*ACE2/ace2-A7*	Inbred diploid resulting from crossing between JAY1529 x JAY1553	This study
JAY1656	*MATα/MATa*	*ACE2/ace2-A7*	Inbred diploid resulting from crossing between JAY1529 x JAY1557	This study
JAY1658	*MATα/MATa*	*ACE2/ace2-A7*	Inbred diploid resulting from crossing between JAY1529 x JAY290	This study
JAY1660	*MATα/MATa*	*ACE2/ace2-A7*	Inbred diploid resulting from crossing between JAY1529 x JAY293	This study
JAY1662	*MATα/MATa*	*ACE2/ace2-A7*	Inbred diploid resulting from crossing between JAY1529 x JAY328	This study
JAY1664	*MATα/MATa*	*ACE2/ace2-A7*	Inbred diploid resulting from crossing between JAY1533 x JAY1536	This study
JAY1666	*MATα/MATa*	*ACE2/ace2-A7*	Inbred diploid resulting from crossing between JAY1533 x JAY1539	This study
JAY1668	*MATα/MATa*	*ACE2/ace2-A7*	Inbred diploid resulting from crossing between JAY1533 x JAY1545	This study
JAY1670	*MATα/MATa*	*ACE2/ace2-A7*	Inbred diploid resulting from crossing between JAY1533 x JAY1546	This study
JAY1672	*MATα/MATa*	*ACE2/ace2-A7*	Inbred diploid resulting from crossing between JAY1533 x JAY1553	This study
JAY1674	*MATα/MATa*	*ACE2/ace2-A7*	Inbred diploid resulting from crossing between JAY1533 x JAY1557	This study
JAY1676	*MATα/MATa*	*ACE2/ace2-A7*	Inbred diploid resulting from crossing between JAY1533 x JAY290	This study
JAY1678	*MATα/MATa*	*ACE2/ace2-A7*	Inbred diploid resulting from crossing between JAY1533 x JAY293	This study
JAY1680	*MATα/MATa*	*ACE2/ace2-A7*	Inbred diploid resulting from crossing between JAY1533 x JAY328	This study
JAY1682	*MATα/MATa*	*ACE2/ace2-A7*	Inbred diploid resulting from crossing between JAY1535 x JAY1539	This study
JAY1684	*MATα/MATa*	*ACE2/ace2-A7*	Inbred diploid resulting from crossing between JAY1535 x JAY1545	This study
JAY1686	*MATα/MATa*	*ACE2/ace2-A7*	Inbred diploid resulting from crossing between JAY1535 x JAY1546	This study
JAY1688	*MATα/MATa*	*ACE2/ace2-A7*	Inbred diploid resulting from crossing between JAY1535 x JAY1553	This study
JAY1690	*MATα/MATa*	*ACE2/ace2-A7*	Inbred diploid resulting from crossing between JAY1535 x JAY1557	This study
JAY1692	*MATα/MATa*	*ACE2/ace2-A7*	Inbred diploid resulting from crossing between JAY1535 x JAY290	This study
JAY1694	*MATα/MATa*	*ACE2/ace2-A7*	Inbred diploid resulting from crossing between JAY1535 x JAY293	This study
JAY1696	*MATα/MATa*	*ACE2/ace2-A7*	Inbred diploid resulting from crossing between JAY1535 x JAY328	This study
JAY1698	*MATα/MATa*	*ACE2/ace2-A7*	Inbred diploid resulting from crossing between JAY1540 x JAY1545	This study
JAY1700	*MATα/MATa*	*ACE2/ace2-A7*	Inbred diploid resulting from crossing between JAY1540 x JAY1546	This study
JAY1702	*MATα/MATa*	*ACE2/ace2-A7*	Inbred diploid resulting from crossing between JAY1540 x JAY1553	This study
JAY1704	*MATα/MATa*	*ACE2/ace2-A7*	Inbred diploid resulting from crossing between JAY1540 x JAY1557	This study

Table S1 (continued). Yeast strains used in this study.

Strain	Mating type	Relevant genotype	Description	Source
JAY1706	*MATα/MATa*	*ACE2/ace2-A7*	Inbred diploid resulting from crossing between JAY1540 x JAY290	This study
JAY1708	*MATα/MATa*	*ACE2/ace2-A7*	Inbred diploid resulting from crossing between JAY1540 x JAY293	This study
JAY1710	*MATα/MATa*	*ACE2/ace2-A7*	Inbred diploid resulting from crossing between JAY1540 x JAY328	This study
JAY1712	*MATα/MATa*	*ACE2/ace2-A7*	Inbred diploid resulting from crossing between JAY1543 x JAY1546	This study
JAY1714	*MATα/MATa*	*ACE2/ace2-A7*	Inbred diploid resulting from crossing between JAY1543 x JAY1553	This study
JAY1716	*MATα/MATa*	*ACE2/ace2-A7*	Inbred diploid resulting from crossing between JAY1543 x JAY1557	This study
JAY1718	*MATα/MATa*	*ACE2/ace2-A7*	Inbred diploid resulting from crossing between JAY1543 x JAY290	This study
JAY1720	*MATα/MATa*	*ACE2/ace2-A7*	Inbred diploid resulting from crossing between JAY1543 x JAY293	This study
JAY1722	*MATα/MATa*	*ACE2/ace2-A7*	Inbred diploid resulting from crossing between JAY1543 x JAY328	This study
JAY1724	*MATα/MATa*	*ACE2/ace2-A7*	Inbred diploid resulting from crossing between JAY1549 x JAY1553	This study
JAY1726	*MATα/MATa*	*ACE2/ace2-A7*	Inbred diploid resulting from crossing between JAY1549 x JAY1557	This study
JAY1728	*MATα/MATa*	*ACE2/ace2-A7*	Inbred diploid resulting from crossing between JAY1549 x JAY290	This study
JAY1730	*MATα/MATa*	*ACE2/ace2-A7*	Inbred diploid resulting from crossing between JAY1549 x JAY293	This study
JAY1732	*MATα/MATa*	*ACE2/ace2-A7*	Inbred diploid resulting from crossing between JAY1549 x JAY328	This study
JAY1734	*MATα/MATa*	*ACE2/ace2-A7*	Inbred diploid resulting from crossing between JAY1552 x JAY1557	This study
JAY1736	*MATα/MATa*	*ACE2/ace2-A7*	Inbred diploid resulting from crossing between JAY1552 x JAY290	This study
JAY1738	*MATα/MATa*	*ACE2/ace2-A7*	Inbred diploid resulting from crossing between JAY1552 x JAY293	This study
JAY1740	*MATα/MATa*	*ACE2/ace2-A7*	Inbred diploid resulting from crossing between JAY1552 x JAY328	This study
JAY1742	*MATα/MATa*	*ACE2/ace2-A7*	Inbred diploid resulting from crossing between JAY1554 x JAY290	This study
JAY1744	*MATα/ MATa*	*ACE2/ace2-A7*	Inbred diploid resulting from crossing between JAY1554 x JAY293	This study
JAY1746	*MATα/MATa*	*ACE2/ace2-A7*	Inbred diploid resulting from crossing between JAY1554 x JAY328	This study
JAY1748	*MATα/MATa*	*ACE2/ace2-A7*	Inbred diploid resulting from crossing between JAY291 x JAY293	This study
JAY1750	*MATα/MATa*	*ACE2/ace2-A7*	Inbred diploid resulting from crossing between JAY291 x JAY328	This study
JAY1752	*MATα/MATa*	*ACE2/ace2-A7*	Inbred diploid resulting from crossing between JAY295 x JAY328	This study
JAY2108	*MATα/MATa*	*ACE2/ace2-A7;* Chr4-UPD P/P	JAY270 derivative containing uniparental disomy for Chr4 (P/P)	This study
JAY2133	*MATα/MATa*	*ACE2/ace2-A7;* Chr4-UPD P/P	JAY270 derivative containing uniparental disomy for Chr4 (P/P)	This study
JAY2191	*MATα/MATa*	*ACE2/ace2-A7;* Chr4-UPD M/M	JAY270 derivative containing uniparental disomy for Chr4 (P/P)	This study
JAY2200	*MATα/MATa*	*ACE2/ace2-A7;* Chr4-UPD P/P	JAY270 derivative containing uniparental disomy for Chr4 (P/P)	This study
JAY2201	*MATα/MATa*	*ACE2/ace2-A7;* Chr4-UPD M/M	JAY270 derivative containing uniparental disomy for Chr4 (M/M)	This study
JAY2202	*MATα/MATa*	*ACE2/ace2-A7;* Chr4-UPD M/M	JAY270 derivative containing uniparental disomy for Chr4 (M/M)	This study
JAY2250	*MATα/MATa*	*ACE2/ace2-A7;* Chr14-UPD P/P	JAY270 derivative containing uniparental disomy for Chr14 (P/P)	This study
JAY2252	*MATα/MATa*	*ACE2/ace2-A7;* Chr14-UPD P/P	JAY270 derivative containing uniparental disomy for Chr14 (P/P)	This study
JAY2254	*MATα/MATa*	*ACE2/ace2-A7;* Chr14-UPD P/P	JAY270 derivative containing uniparental disomy for Chr14 (P/P)	This study
JAY2244	*MATα/MATa*	*ACE2/ace2-A7;* Chr14-UPD M/M	JAY270 derivative containing uniparental disomy for Chr14 (M/M)	This study
JAY2248	*MATα/MATa*	*ACE2/ace2-A7;* Chr14-UPD M/M	JAY270 derivative containing uniparental disomy for Chr14 (M/M)	This study
JAY2282	*MATα/MATa*	*ACE2/ace2-A7;* Chr15-UPD P/P	JAY270 derivative containing uniparental disomy for Chr15 (P/P)	This study
JAY2283	*MATα/MATa*	*ACE2/ace2-A7;* Chr15-UPD P/P	JAY270 derivative containing uniparental disomy for Chr15 (P/P)	This study
JAY2284	*MATα/MATa*	*ACE2/ace2-A7;* Chr15-UPD P/P	JAY270 derivative containing uniparental disomy for Chr15 (P/P)	This study
JAY2298	*MATα/MATa*	*ACE2/ace2-A7;* Chr15-UPD M/M	JAY270 derivative containing uniparental disomy for Chr15 (M/M)	This study
JAY2300	*MATα/MATa*	*ACE2/ace2-A7;* Chr15-UPD M/M	JAY270 derivative containing uniparental disomy for Chr15 (M/M)	This study

Table S2. Oligonucleotides used in this study.

Name	5' – 3' sequence [a]	Description
JAO14	**AGGAGGGTATTCTGGGCCTCCATG**	Inside *MX4* region. To confirm insertion of *MX4* cassettes; Rev.
JAO15	**ATGCGAAGTTAAGTGCGCAGAAAG**	Inside *MX4* region. To confirm insertion of *MX4* cassettes; Fwd.
JAO1385	**GATTACACCGTACTTCTTTTCAATGCGTAAACAACTAGAGTTGACAAA** CTGCTGTCGATTCGATAC	To amplify *GFP* cassette; Fwd. Homology tail to *CEN5* region.
JAO1386	**CACGTCAAGACTGTCAAGGAGGGTATTCTGGGCCTCCATGTC** GTCGATGAATTCGAGCTC	To amplify *GFP* cassette; Rev. Homology tail to *KanMX4* cassette.
JAO1387	GACATGGAGGCCCAGAATAC	To amplify *KanMX4 cassette;* Fwd.
JAO466	**TACGAAGTGCTAGGAGGTATATTATTAATATGATAGTTTTAGTTTATAATA** GTGGATCTGATATCATCG	To amplify *KanMX4* cassette; Rev. Homology tail to *CEN5* region.
JAO464	CATCGTGTAGTCAAGCAGCA	To confirm integration of *GFP-KanMX4* cassette; Fwd. *CEN5* region.
JAO467	CATTTACAGATTCATAGTTC	To confirm integration of *GFP-KanMX4* cassette; Rev. *CEN5* region.
JAO1563	**ACAGTACTAGCTTTTAACTTGTATCCTAGGTTATCTATGCTGTCTCACCATAGGGAATAT TACCTATTTCAG** CTTGCCTCGTCCCCGCCG	To amplify *AmdS* cassette; Fwd. Homology tail to *CEN4* region.
JAO1566	**GTATTTTAAGTTTTTAAAAAAGTTGATTAAATAGCATGTGAC** CTCCAGTATAGCGACCAG	To amplify *AmdS* cassette; Rev. Homology tail to region immediately upstream to *CEN4*.
JAO1567	**AAAAAGTATTTTAAGTTTTTAAAAAAGTTGATTAAATAGCAT** CTCCAGTATAGCGACCAG	To amplify *AmdS* cassette; Rev. Integration results in 5bp deletion of *CEN4* element 1.
JAO1568	**GTATTTTAAGTTTTTAAAAAAGTTGATTAAATAGCATGTGAC** AGTTATGGAGTAACAACG	To amplify *AmdS* cassette excluding its terminator region; Rev. Homology tail to region immediately upstream to *CEN4*.
JAO1569	CTGCAAAACAGTACTAGCTTTTAACTTG	To confirm integration of *AmdS* at *CEN4* region; Fwd.
JAO1570	GGAATATATAGCAGTAGTCAATTTAGCAC	To confirm integration of *AmdS* at *CEN4* region; Rev. Used for Sanger sequencing.
JAO1574	TCCAAGGTGGTTGCATCATA	Chr4 710,025 SNP *Bgl*II RFLP marker Fwd
JAO1575	TGGCTGGAGTTTCGTCTTCT	Chr4 710,025 SNP *Bgl*II RFLP marker Rev
JAO1576	GTATCTTAATGAAACTATGCAATGG	Chr4 378,184 SNP *Bam*HI RFLP marker Fwd
JAO1577	CCTCATCGGCACATTAAAGCTG	Chr4 378,184 SNP *Bam*HI RFLP marker Rev
JAO1659	**AAAGAAAAAAATTACTGCAAAACAGTACTAGCTTTTAACTTGTATCCTAGGTTATCTATG CTGTCTCACCAT** CTTGCCTCGTCCCCGCCG	To amplify *AmdS* cassette; Fwd. Homology tail to *CEN4* region
JAO1681	**TGGAGGAAAAGCATTGGTATTAAGTACTTTGCATTCTCTTGGAGAAGAACTTGATCAATT GACGGGTATAGC** CTTGCCTCGTCCCCGCCG	To amplify *AmdS* cassette; Fwd. Homology tail to *CEN14* region
JAO1682	**AAAATGTTTTAAAATATTTTTAAAAAGCTGCACGTGACTAAC** AGTTATGGAGTAACAACG	To amplify *AmdS* cassette excluding its terminator region; Rev. Homology tail to region immediately upstream to *CEN14*.
JAO1683	GGACTACTGATGTACTGAAGTTTG	To confirm integration of *AmdS* at *CEN14* region; Fwd
JAO1684	CCTGCTGTCTAATCCGTATTCATTC	To confirm integration of *AmdS* at *CEN14* region; Rev
JAO1685	CTGAATTGGACGCTTTGGTTC	Inside *AmdS* gene. To confirm insertion of *AmdS* cassettes; Fwd.
JAO1686	**TCATCACTGTTCTTTTCAGATACTAGTTTCAAAAATTCCTTGACAGAACCATTTCATGTTC AATAATGAAAA** CTTGCCTCGTCCCCGCCG	To amplify *AmdS* cassette; Fwd. Homology tail to *CEN15* region
JAO1687	**AATGTTTAAATATTTAATGTATATGACTTCCGAAAAATATAT** AGTTATGGAGTAACAACG	To amplify *AmdS* cassette excluding its terminator region; Rev. Homology tail to region immediately upstream to *CEN15*.
JAO1688	GGTAGTATATAAGAGAATAACTTCCC	To confirm integration of *AmdS* at *CEN15* region; Fwd
JAO1689	GCTCTCACCACATTTATCACC	To confirm integration of *AmdS* at *CEN15* region; Rev
JAO1692	CACAAAATATGTTCGTACCCATCC	Chr14 259,198 SNP *Mfe*II RFLP marker Fwd
JAO1693	GTGACGGAGTTTGATATACTTAAC	Chr14 259,198 SNP *Mfe*II RFLP marker Rev

Table S2 (continued). Oligonucleotides used in this study.

Name	5' – 3' sequence [a]	Description
JAO1694	GTTGAAGCAATGAAGGAATCTTCG	Chr14 758,547 SNP *Mse*I RFLP marker Fwd
JAO1695	CCTCAGTCATGCCAAGTGCATCAAC	Chr14 758,547 SNP *Mse*I RFLP marker Rev
JAO1696	CGTCTCGTATTATACTTGTCCTG	Chr15 16,733 SNP *Sal*I RFLP marker Fwd
JAO1697	CATCGAGGAACAACAGCATCTAG	Chr15 16,733 SNP *Sal*I RFLP marker Rev
JAO1983	CAATAATTCTCCTAGTGTTGTGC	Chr15 130,295 SNP *Pst*I RFLP marker Fwd
JAO1984	CTGGTGTAAAGGCGTACTGAAC	Chr15 130,295 SNP *Pst*I RFLP marker Rev
JAO1588	GAGTTATTGACAGGGAATAC	Chr15 1,058,946 SNP *Nde*I RFLP marker Fwd
JAO1589	GGAGCTTACTTCACTCATTTC	Chr15 1,058,946 SNP *Nde*I RFLP marker Rev
JAO1063	AGTCACATCAAGATCGTTTATGG	Chr3, mating type reverse primer; to amplify *MATa* and/or *MAT*α
JAO1064	GCACGGAATATGGGACTACTTCG	Chr3, *MAT*α forward primer; to amplify *MAT*α
JAO1065	ACTCCACTTCAAGTAAGAGTTTG	Chr3, *MATa* forward primer; to amplify *MATa*

a. Nucleotides in the 5' end (**bold**) have homology to the respective chromosomal insertion sites. 3' end nucleotides (not in bold) correspond to primer sequences to amplify the desired amplicons.

Table S3. Growth conditions used in phenotypic screening of inbred strain collection through plate spotting assay.

Base Media	Additional treatment/supplement	Temperature of incubation	Hours of incubation
2% YPD	-	30C	48
2% YPD	-	39C	96
2% YPGE	-	30C	48
2% YP Galactose	-	30C	48
2% YP Raffinose	-	30C	48
2% YPD	7 % Ethanol	30C	48
2% YPD	11 % Ethanol	30C	48
2% YPD	30 mM furfural	30C	48
2% YPD	0.75 mM menadione	30C	48
2% YPD	0.01% MMS	30C	48
2% YPD	100 mM hydroxyurea	30C	96
2% YPD	100 J/m^2 UV light	30C	48
2% YPD	150 J/m^2 UV light	30C	48

Table S4. Phenotypic data of inbreds from competition assays (CA) and tolerance to high temperature assays (HT).

Strain	[a]Mean % inbred cells in the population in CA				[b]Median Score in HT
	Cycle 0	Cycle 2	Cycle 5	Cycle 8	
JAY1598	50.50 ± 0.40	33.72 ± 0.41	19.15 ± 0.17	13.32 ± 0.49	3.50 (0.42)
JAY1600	54.40 ± 0.31	36.87 ± 0.12	22.27 ± 0.16	10.03 ± 0.19	1.00 (0.46)
JAY1602	50.65 ± 0.26	18.00 ± 0.18	5.72 ± 0.27	2.39 ± 0.06	3.50 (0.28)
JAY1604	54.45 ± 0.19	38.85 ± 0.30	25.68 ± 0.27	12.97 ± 0.26	1.00 (0.43)
JAY1606	54.00 ± 0.10	59.88 ± 0.40	74.95 ± 0.19	78.28 ± 0.34	1.00 (0.25)
JAY1608	54.10 ± 0.41	43.32 ± 0.15	32.38 ± 0.35	26.13 ± 0.30	3.25 (0.25)
JAY1610	54.20 ± 0.13	39.92 ± 0.40	37.22 ± 0.39	25.55 ± 0.70	5.00 (0.00)
JAY1612	49.95 ± 0.30	36.57 ± 0.28	25.97 ± 0.50	14.63 ± 0.32	4.00 (0.31)
JAY1614	47.15 ± 0.09	11.48 ± 0.14	1.27 ± 0.07	0.81 ± 0.07	3.50 (0.28)
JAY1616	50.35 ± 0.38	57.08 ± 0.54	73.27 ± 0.41	70.05 ± 0.26	3.00 (0.26)
JAY1618	53.55 ± 0.08	57.93 ± 0.32	73.47 ± 0.15	76.57 ± 0.61	1.00 (0.39)
JAY1620	47.45 ± 0.21	22.00 ± 0.06	6.85 ± 0.17	3.11 ± 0.14	4.00 (0.62)
JAY1622	49.75 ± 0.18	52.28 ± 0.15	62.38 ± 0.48	59.13 ± 0.30	0.00 (0.26)
JAY1624	47.95 ± 0.26	38.35 ± 0.43	31.53 ± 0.45	21.43 ± 0.51	3.00 (0.26)
JAY1626	48.25 ± 0.20	53.23 ± 0.74	69.08 ± 0.25	76.03 ± 0.24	1.50 (0.42)
JAY1628	47.70 ± 0.40	67.25 ± 0.46	92.93 ± 0.16	98.15 ± 0.12	2.00 (0.00)
JAY1630	47.60 ± 0.27	50.40 ± 0.64	64.62 ± 0.71	71.98 ± 0.22	3.00 (0.26)
JAY1632	48.75 ± 0.32	48.18 ± 0.79	63.85 ± 0.31	70.22 ± 0.17	5.00 (0.42)
JAY1634	45.95 ± 0.48	46.48 ± 0.91	51.25 ± 0.52	51.07 ± 0.39	3.00 (0.41)
JAY1636	49.05 ± 0.32	33.93 ± 0.99	16.63 ± 0.34	7.22 ± 0.70	3.00 (0.63)
JAY1638	46.60 ± 1.87	58.07 ± 0.44	83.22 ± 0.50	93.55 ± 0.37	2.25 (0.52)
JAY1640	47.40 ± 0.74	63.05 ± 0.98	90.82 ± 0.14	97.47 ± 0.10	1.00 (0.43)
JAY1642	40.25 ± 1.59	28.00 ± 0.82	24.68 ± 0.81	15.60 ± 0.63	3.50 (0.13)
JAY1644	47.95 ± 0.39	45.53 ± 1.02	62.88 ± 0.31	61.22 ± 0.78	4.00 (0.21)
JAY1646	49.20 ± 0.90	55.67 ± 0.72	79.45 ± 0.28	88.7 ± 0.50	3.25 (0.25)
JAY1648	45.40 ± 0.44	65.70 ± 1.03	93.83 ± 0.24	97.42 ± 0.05	4.00 (0.21)
JAY1650	49.45 ± 0.93	46.10 ± 0.72	68.23 ± 1.57	70.28 ± 1.85	4.25 (0.21)

a. Mean value between 3 replicates plus or minus standard error.
b. Median value between 3 replicates. Standard error of the median indicated in parentheses and calculated by multiplying standard error of mean by 1.253.

Table S4 (continued). Phenotypic data of inbreds from competition assays (CA) and tolerance to high temperature assays (HT).

Strain	[a]Mean % inbred cells in the population in CA				[b]Median Score in HT
	Cycle 0	Cycle 2	Cycle 5	Cycle 8	
JAY1652	45.75 ± 0.47	54.32 ± 0.77	81.95 ± 0.74	91.70 ± 0.60	4.25 (0.91)
JAY1654	39.90 ± 1.48	61.55 ± 0.73	88.72 ± 0.42	96.30 ± 0.20	4.00 (0.13)
JAY1656	45.40 ± 0.73	44.32 ± 0.85	49.82 ± 2.32	50.20 ± 3.18	4.25 (0.39)
JAY1658	45.35 ± 0.63	65.97 ± 0.82	90.13 ± 0.33	96.32 ± 0.24	4.00 (0.00)
JAY1660	49.60 ± 0.93	64.93 ± 0.47	87.97 ± 0.53	96.30 ± 0.13	4.00 (0.26)
JAY1662	40.60 ± 0.88	30.02 ± 0.95	20.12 ± 1.01	12.95 ± 0.99	4.00 (0.26)
JAY1664	47.05 ± 0.69	67.30 ± 1.42	92.93 ± 0.49	97.93 ± 0.20	0.00 (0.00)
JAY1666	51.83 ± 0.42	41.30 ± 0.85	28.87 ± 0.30	18.93 ± 0.39	0.00 (0.00)
JAY1668	52.20 ± 0.58	28.53 ± 0.56	5.56 ± 0.46	2.29 ± 0.31	2.50 (0.75)
JAY1670	52.02 ± 0.42	63.97 ± 0.81	71.73 ± 0.68	74.48 ± 0.56	3.00 (0.10)
JAY1672	50.53 ± 0.36	51.68 ± 0.99	45.70 ± 0.93	25.42 ± 0.72	0.50 (0.42)
JAY1674	49.07 ± 0.40	37.38 ± 0.49	18.28 ± 0.53	9.05 ± 0.32	3.00 (0.13)
JAY1676	50.70 ± 0.48	51.02 ± 1.44	45.40 ± 0.58	29.93 ± 0.55	0.50 (0.42)
JAY1678	49.55 ± 0.20	41.27 ± 1.36	50.87 ± 0.58	46.52 ± 0.79	1.00 (0.39)
JAY1680	45.35 ± 0.47	59.17 ± 0.39	28.92 ± 1.55	38.80 ± 0.58	3.00 (0.21)
JAY1682	50.23 ± 0.71	45.62 ± 1.01	47.33 ± 0.62	34.42 ± 0.52	3.00 (0.42)
JAY1684	49.00 ± 0.31	36.33 ± 0.68	14.77 ± 0.68	5.20 ± 0.36	4.00 (0.26)
JAY1686	52.93 ± 0.42	31.18 ± 0.96	28.03 ± 0.29	17.82 ± 0.52	4.00 (0.26)
JAY1688	52.82 ± 0.42	30.47 ± 1.46	24.83 ± 0.45	14.92 ± 0.22	5.00 (0.00)
JAY1690	49.32 ± 0.37	28.15 ± 0.42	8.07 ± 0.38	2.89 ± 0.29	5.00 (0.13)
JAY1692	43.13 ± 0.34	32.80 ± 1.55	24.40 ± 1.12	15.27 ± 0.74	4.00 (0.00)
JAY1694	48.32 ± 0.67	30.17 ± 1.76	27.12 ± 1.13	14.50 ± 1.14	3.00 (0.00)
JAY1696	44.93 ± 0.49	44.48 ± 0.98	31.73 ± 1.56	33.77 ± 0.86	4.25 (0.25)
JAY1698	52.42 ± 0.53	48.17 ± 1.22	31.33 ± 1.40	16.68 ± 0.68	3.00 (0.00)
JAY1700	48.68 ± 0.31	32.05 ± 1.64	26.65 ± 0.45	2.93 ± 0.22	5.00 (0.00)
JAY1702	50.48 ± 0.73	30.12 ± 1.45	17.13 ± 0.34	2.96 ± 0.46	2.50 (0.51)

a. Mean value between 3 replicates plus or minus standard error.
b. Median value between 3 replicates. Standard error of the median indicated in parentheses and calculated by multiplying standard error of mean by 1.253.

Table S4 (continued). Phenotypic data of inbreds from competition assays (CA) and tolerance to high temperature assays (HT).

Strain	[a]Mean % inbred cells in the population in CA				[b]Median Score in HT
	Cycle 0	Cycle 2	Cycle 5	Cycle 8	
JAY1704	49.87 ± 0.45	55.72 ± 0.49	71.50 ± 0.42	84.67 ± 0.30	4.50 (0.38)
JAY1706	50.80 ± 0.96	64.72 ± 0.45	86.90 ± 0.29	96.1 ± 0.41	3.50 (0.13)
JAY1708	53.53 ± 0.48	81.92 ± 0.25	92.57 ± 0.25	97.25 ± 0.21	3.00 (0.26)
JAY1710	46.03 ± 0.37	25.37 ± 0.54	25.25 ± 0.79	24.93 ± 1.63	5.00 (0.21)
JAY1712	51.80 ± 0.32	19.22 ± 0.67	16.70 ± 0.50	16.70 ± 0.72	4.00 (0.00)
JAY1714	52.30 ± 0.34	24.70 ± 0.54	22.67 ± 0.60	23.55 ± 0.37	3.00 (0.63)
JAY1716	51.90 ± 0.22	7.05 ± 0.32	3.56 ± 0.51	4.00 ± 0.56	2.50 (0.65)
JAY1718	51.47 ± 0.27	46.05 ± 0.35	57.65 ± 0.55	75.33 ± 0.87	2.00 (0.52)
JAY1720	49.82 ± 0.28	41.17 ± 1.24	43.68 ± 1.19	35.63 ± 1.01	1.50 (0.54)
JAY1722	46.68 ± 0.39	7.64 ± 0.76	5.25 ± 0.38	4.94 ± 0.53	3.00 (0.00)
JAY1724	43.30 ± 0.75	18.58 ± 0.55	14.02 ± 0.42	8.49 ± 0.40	1.50 (0.28)
JAY1726	43.73 ± 0.59	8.52 ± 0.35	4.18 ± 0.43	5.11 ± 0.45	4.00 (0.13)
JAY1728	39.18 ± 0.94	34.82 ± 2.23	25.07 ± 1.19	16.42 ± 0.36	3.00 (0.26)
JAY1730	40.37 ± 0.27	33.60 ± 2.30	23.30 ± 0.78	17.52 ± 1.46	1.00 (0.26)
JAY1732	38.78 ± 2.23	13.20 ± 0.85	4.97 ± 0.73	1.68 ± 0.17	3.00 (0.26)
JAY1734	51.42 ± 0.35	36.17 ± 0.54	12.00 ± 0.40	3.91 ± 0.49	4.00 (0.21)
JAY1736	50.88 ± 0.33	59.33 ± 0.47	69.98 ± 0.49	79.22 ± 0.42	3.00 (0.00)
JAY1738	51.72 ± 0.35	55.88 ± 0.70	58.42 ± 1.26	61.85 ± 1.41	3.00 (0.26)
JAY1740	47.28 ± 0.35	35.82 ± 0.53	19.05 ± 0.62	10.22 ± 0.56	4.00 (0.00)
JAY1742	53.95 ± 0.25	69.72 ± 0.25	90.65 ± 0.28	97.78 ± 0.10	1.50 (0.28)
JAY1744	55.13 ± 0.27	65.48 ± 0.32	86.57 ± 0.46	95.35 ± 0.05	0.50 (0.36)
JAY1746	48.22 ± 0.24	42.45 ± 0.53	41.25 ± 0.61	38.33 ± 0.80	3.00 (0.13)
JAY1748	51.38 ± 0.38	38.30 ± 0.33	22.48 ± 0.18	12.52 ± 0.36	2.50 (0.23)
JAY1750	48.55 ± 0.59	15.60 ± 0.43	4.05 ± 0.47	1.83 ± 0.33	4.00 (0.13)
JAY1752	48.17 ± 0.81	15.15 ± 0.13	4.11 ± 0.49	2.31 ± 0.50	3.00 (0.13)

a. Mean value between 3 replicates plus or minus standard error.
b. Median value between 3 replicates. Standard error of the median indicated in parentheses and calculated by multiplying standard error of mean by 1.253.

Table S5. List of phased JAY270 HetSNPs interrogated for confirmation of UPD strains.

Marker SGD coordinates		PCR primers	Polymorphism detection method	[a]SGD Watson bases	JAY270 Watson phased bases	
**Chromosome**	**Nucleotide**				**Chr-P**	**Chr-M**
Chr4	378,184	JAO1576/JAO1577	*BamH*I	C	T	C
Chr4	710,025	JAO1574/JAO1575	*Bgl*II	T	G	T
Chr14	259,198	JAO1692/JAO1693	*Mfe*I	T	T	A
Chr14	758,547	JAO1694/JAO1695	*Mse*I	A	G	A
Chr15	16,733	JAO1696/JAO1697	*Sal*I	C	T	C
Chr15	130,295	JAO1983/JAO1984	*Pst*I	A	A	C
Chr15	1,058,946	JAO1588/JAO1589	*Nde*I	T	T	C

a. Nucleotides that are underlined indicate the sequences that are cut by the respective restriction endonucleases, whereas lack of underlining corresponds to no cut site.

Table S6. Regions showing significant association to the heat tolerance and competitive growth phenotypes and their gene contents.

Phenotype	Chr	Genome Coordinates	HetSNPs	Max LOD	Total Genes	Genes with HetSNPs	Genes with Nonsynonymous Substitution(s)	List of Genes with Nonsynonymous Substitution(s)
Heat Tolerance	2	Chr02_207698..214801 (7.1 Kb)	14	4.45	2	2	1	*YBL008W-A*
	5	Chr05_549677..565641 (16.0 Kb)	40	5.47	9	2	2	*ISC10*, *SLO1*
	6	Chr06_74814..97759 (22.9 Kb)	91	6.23	14	13	8	*HAC1*, *CAF16*, *GYP8*, *STE2*, *BST1*, *BUD27*, *FRS2*, *GAT1*
	7	Chr07_82753..88718 (6.0 Kb)	11	4.61	4	2	1	*KIP3*
	7	Chr07_673392..730469 (57.1 Kb)	9	7.12	30	2	0	none
	7	Chr07_849524..887971 (38.4 Kb)	14	5.56	20	3	0	none
	12	Chr12_176348..201613 (25.3 Kb)	88	4.28	14	10	6	*PRS2*, *YEH2*, *IRC25*, *SDO1*, *UBR2*, *ADE16*
	13	Chr13_75040..92531 (17.5 Kb)	49	8.67	12	9	4	*TAF13*, *YML096W*, *UTP14*, *RPM2*
	13	Chr13_715996..749967 (34.0 Kb)	13	8.70	19	5	2	*MRE11*, *BCH1*
	13	Chr13_754723..814438 (59.7 Kb)	59	5.20	37	19	13	*YMR244W*, *YMR253C*, *GFD1*, *PET111*, *TRM732*, *TPS3*, *RSN1*, *PPA2*, *PRP24*, *RRN9*, *URA10*, *SCS7*, *ZDS1*
	14	Chr14_528514..605480 (77.0 Kb)	66	11.06	47	14	9 (1 nonsense)	*COG5*, *SFB2*, *ALG11*, *SLM2* (nonsense), *YNL034W*, *KTR5*, *CRZ1*, *SAM50*, *FAP1*
	14	Chr14_691878..695118 (3.2 Kb)	6	5.26	3	1	0	none
	15	Chr15_764174..781222 (17.0 Kb)	13	6.01	10	1	1	*YOR228C*
Competitive Growth	3	Chr03_77849..84690 (6.8 Kb)	44	4.34	5	3	2	*KCC4*, *YCL021W-A*
	4	Chr04_556996..592486 (35.5 Kb)	99	6.00	24	18	11	*DBF4*, *PST1*, *MAK21*, *YDR061W*, *LCB2*, *AIM7*, *RRG1*, *RRTR2*, *DOS2*, *DOA4*, *IPT1*
	8	Chr08_35149..51314 (16.2 Kb)	26	4.68	7	2	2	*ECM29*, *WSC4*
	10	Chr10_467589..569699 (102.1 Kb)	48	8.30	56	17	8	*REC107*, *YJR030C*, *GEA1*, *CPR7*, *RAD26*, *URB2*, *TOR1*, *YAE1*
	15	Chr15_882911..889659 (6.7 Kb)	3	4.13	2	1	0	none
	15	Chr15_1024820..1069385 (44.6 Kb)	138	4.31	23	12	10	*YOR365C*, *GFB1*, *NDD1*, *NUD1*, *YOR376W-A*, *ATF1*, *FRE3*, *YOR381W-A*, *YOR385W*, *PHR1*
	16	Chr16_289873..361102 (71.2 Kb)	134	5.93	40	22	15	*SPP1*, *GIP3*, *ISU1*, *COX11*, *SPO19*, *TBF1*, *HHO1*, *NAN1*, *KAP120*, *MRP51*, *HOS3*, *CAR1*, *YPL109C*, *YPL108W*, *FMP30*

Table S7. Single locus analysis of variance for heat tolerance and competitive growth phenotypes in the inbred strains.

Phenotype	Locus	Significance	[a]PVE	[b]Relative PVE	Inheritance model	Parent Contributing high value allele
Heat Tolerance	Chr02_209988	<0.0001	23.12	0.48	Additive	P
	Chr05_564935	<0.0001	27.59	0.58	Dominant	P
	Chr06_87106	<0.0001	30.77	0.64	Additive	M
	Chr07_86432	<0.0001	23.82	0.50	Additive	M
	Chr07_677969	<0.0001	34.34	0.72	Additive	P
	Chr07_858098	<0.0001	27.99	0.58	Additive	P
	Chr12_188842	<0.0001	22.31	0.47	Dominant	P
	Chr13_83905	<0.0001	40.04	0.84	Additive	M
	Chr13_743742	<0.0001	40.16	0.84	Additive	P
	Chr13_799417	<0.0001	26.45	0.55	Additive	P
	Chr14_572098	<0.0001	47.95	1.00	Additive	M
	Chr14_691878	<0.0001	26.68	0.56	Dominant	M
	Chr15_764348	<0.0001	29.86	0.62	Additive	M
Competitive Growth	Chr03_82057	<0.0001	22.6	0.58	Additive	P
	Chr04_560433	<0.0001	29.82	0.77	Additive	M
	Chr08_49196	<0.0001	24.13	0.62	Additive	P
	Chr10_488369	<0.0001	38.74	1.00	Additive	P
	Chr15_888521	<0.0001	21.64	0.56	Additive	P
	Chr15_1055015	<0.0001	22.47	0.58	Dominant	M
	Chr16_340133	<0.0001	29.52	0.76	Additive	M

a. The PVE values obtained through this single-QTL model are likely over-estimates of the values.
b. PVE values normalized to highest value PVE for each phenotype.

Table S8. Multi-locus QTL models that best explain variation seen in heat tolerance and competitive growth inbred strains.

Phenotype	Locus	Significance	Total Percent Variance Explained
Heat Tolerance	Chr06_87106	<0.0001	75.88
	Chr13_743742	<0.0001	
	Chr14_572098	<0.0001	
Competitive Growth	Chr04_560433	0.0015	57.27
	Chr10_488369	<0.0001	
	Chr15_1055015	0.0047	
